# The Early Asexual Development Regulator *fluG* Codes for a Putative Bifunctional Enzyme

**DOI:** 10.3389/fmicb.2019.00778

**Published:** 2019-04-17

**Authors:** Mikel Iradi-Serrano, Leire Tola-García, Marc S. Cortese, Unai Ugalde

**Affiliations:** Microbial Biochemistry Laboratory, Department of Applied Chemistry, Faculty of Chemistry, University of the Basque Country, San Sebastian, Spain

**Keywords:** FluG, *Aspergillus nidulans*, enzyme, development, amidohydrolase, γ-glutamyl ligase

## Abstract

FluG is a long recognized early regulator of asexual development in *Aspergillus nidulans*. *fluG* null mutants show profuse aerial growth and no conidial production. Initial studies reported sequence homology of FluG with a prokaryotic type I glutamine synthetase, but catalytic activity has not been demonstrated. In this study, we conducted an in-depth analysis of the FluG sequence, which revealed a single polypeptide containing a putative N-terminal amidohydrolase region linked to a putative C-terminal γ-glutamyl ligase region. Each region corresponded, separately and completely, to respective single function bacterial enzymes. Separate expression of these regions confirmed that the C-terminal region was essential for asexual development. The N-terminal region alone did not support conidial development, but contributed to increased conidial production under high nutrient availability. Point mutations directed at respective key catalytic residues in each region demonstrated that they were essential for biological function. Moreover, the substitution of the N- and C-terminal regions with homologs from *Lactobacillus paracasei* and *Pseudomonas aeruginosa*, respectively, maintained functionality, albeit with altered characteristics. Taken together, the results lead us to conclude that FluG is a bifunctional enzyme that participates in an as yet unidentified metabolic or signaling pathway involving a γ-glutamylated intermediate that contributes to developmental fate.

## Introduction

Surface cultures of the model ascomycete *Aspergillus nidulans*, present a complex developmental pattern which combines vegetative hyphae with asexual (Adams et al., 1998) and sexual structures (Dyer and O’Gorman, 2012). This pattern is modulated in response to environmental factors, such as the composition of the substrate, exposure to the gas phase and light.

The genetic regulation that governs the primal transition from vegetative hyphae to asexual development has been examined in considerable detail. A set of genes, which are expressed in vegetative hyphae, are required to initiate the morphogenetic process. They are collectively known as Upstream Developmental Activators (UDAs). These, in turn, activate a second set of the genes, known as the Central Developmental Pathway (CDP), which are only expressed in conidia-bearing structures (Yu et al., 2006).

One of the earliest acting UDA factors is *fluG* (AN4819). Its deletion results in colonies which fail to produce conidiophores and accumulate aerial vegetative hyphae resulting in a raised mycelial morphology, commonly known as *fluffy* (Lee and Adams, 1994). In addition, *ΔfluG* mutants show defects in autolysis (Emri et al., 2005) and secretion (Wang et al., 2015).

A remarkable feature of FluG signaling involves its extracellular transmission. A wild type colony can induce the sporulation of an adjacent *fluG* null mutant separated by a membrane (Lee and Adams, 1994). The search for the signal conducted by Rodriguez-Urra et al. (2012) identified an adduct of two excreted secondary metabolites [diorcinol and dehydroaustinol (DHO)] that was capable of inducing a partial sporulation response when added onto a *fluG* null mutant colony. Interestingly, Márquez-Fernández et al. (2007) had earlier discovered that the deletion of a phosphopantetheinyl transferase (PPTase) *cfwA*/*npgA*, an enzyme that acting upstream of DHO biosynthesis, exhibited a phenotype with severely reduced growth and asexual development. Rodriguez-Urra et al. (2012) pointed out, however, that an earlier report by Nielsen et al. (2011) described the phenotype of a null mutant of the gene coding for the synthesis of the austinol (*ausA*, AN8383), the precursor in the DHO biosynthesis, as conidial. Hence, a clarification on the connection between FluG signaling and DHO-induced conidiation is pending.

Early studies on the FluG protein showed that it is expressed in the cytosol throughout all stages of development, and that the C-terminal region (aa387 to aa865) is necessary for induction of conidiophore development. Interestingly, this region showed sequence similarity with prokaryotic type I glutamine synthetase (GSI) (Lee and Adams, 1994; D’Souza et al., 2001). Later investigations showed, however, that this region did not display GS activity (Margelis et al., 2001). Other reports indicated that it resembles gene SCO6962 from *Streptomyces coelicolor* (Rexer et al., 2006) which has been attributed to be a γ-glutamyl ligase (GGL) (Krysenko et al., 2017). The N-terminal region has no attributed functional role, but has been reported to bear similarity with an amidohydrolase (SCO6961) from *Streptomyces coelicolor* (Rexer et al., 2006).

Although *fluG-*like sequences (containing a N-terminal amidohydrolase and a GSI) have been reported in the Aspergilli (Mah and Yu, 2006; Ogawa et al., 2010; Chang et al., 2012; Wang et al., 2015), they are also present in genomes of diverse fungal genera (Schumacher et al., 2015; Li et al., 2017), bacteria (Strong et al., 2003; Rexer et al., 2006) and higher plants, where the proteins have attributed roles in nodulation and biotic stress signaling (Mathis et al., 1999, 2000; Trevaskis et al., 2002; Doskočilová et al., 2011; Silva et al., 2015).

In this investigation, we confirm that an *ausA* null mutant has a conidiating phenotype, thus showing that FluG-related signaling is not directly connected to DHO-induced conidiation. In addition, an updated structural analysis of the FluG protein revealed that the N-terminal region presented structural similarity to a prokaryotic amidohydrolase from *Lactobacillus paracasei*, and the C-terminal region presented structural similarity to a γ-glutamyl aromatic monoamine ligase from *Pseudomonas aeruginosa*. Alanine substitutions of the predicted key catalytic residues in each region yielded loss of function phenotypes which were comparable to those of the respective null mutants. Finally, the replacement of both N- and C-terminal regions by their respective homologs, yielded functional phenotypes. Our findings situate FluG as a putative bifunctional enzyme that may regulate the balance between vegetative growth and asexual development through the levels of a γ-glutamylated metabolite.

## Materials and Methods

### Fungal Strains and Culture Conditions

The strains used are shown in Supplementary Table 1. Wild type (*veA+*) strains were used for FluG mutagenesis and phenotypic analysis. For the analysis of phenotypic complementation experiments, and *ausA*-related assays, *veA1* strains were used, in order to establish comparisons with previously published experiments (Lee and Adams, 1994; Yager et al., 1998; D’Souza et al., 2001; Nielsen et al., 2011). Strains were cultured as previously described by Pontecorvo et al. (1953) in *Aspergillus* minimal medium (MMA), using trace elements as described by Käfer (1965), or complete medium (CMA; MMA + 5 g/L yeast extract) with a pH 6.8. Ammonium tartrate (5 mM) and D-glucose (2% w/v) were added as nitrogen and carbon sources, respectively, in solid or liquid form with the appropriate supplements. All cultures where incubated at 37°C in white light (Sera DayLight Brilliant T8 15 W/m^2^; light source at 30 cm from the cultures). Phenotypic complementation experiments were carried out in CMA medium, incubating for a further 72 h after the strains contacted each other (Lee and Adams, 1994). In fluorescence microscopy experiments, strains were grown in adequately supplemented MMA containing 0.1% D-glucose, 5 mM ammonium tartrate and 25 mM monobasic sodium phosphate, similar to watch minimal medium (WMM) (Peñalva, 2005).

### Generation of Mutant, Tagged, and Overexpression Strains

The oligonucleotides used are listed in Supplementary Table 2. Genomic cassettes were amplified through the fusion-PCR technique (Yang et al., 2004; Korbie and Mattick, 2008). The *A. nidulans* transformation technique used was an adaptation of the protoplast generation procedure described by Szewczyk et al. (2006), followed by the transformation protocol described by Tilburn et al. (1983). The strains coding for separate regions of FluG, point mutants of both regions and homologous regions of *Lactobacillus paracasei* and *Pseudomonas aeruginosa* genes were generated by transforming protoplasts of a *ΔfluG* strain with the genomic cassettes bearing the desired modifications plus the 5′- and 3′-UTR regions. The selection of transformants was done using 5-fluoroorotic acid monohydrate (5-FOA; 2 mg/mL; Apollo Scientific, Stockport, United Kingdom).

N- and C-terminally GFP-tagged *fluG* strains were obtained using the method for GFP-tagging described by Yang et al. (2004). The strain carrying the inducible *alcA* expression promoter of the N-terminally GFP-tagged *fluG* was constructed using plasmid pNT5 (Takeshita et al., 2008). *alcA(p)::GFP::fluG* expressing plasmid was obtained by cloning *fluG* (PCR amplified with oligonucleotides AscI_fluG(-ATG)-F and BamHI_fluG-R, 1 kb on the N-terminal sequence of *fluG* with no starting ATG sequence; see Supplementary Table 2) into the AscI and BamHI sites of pNT5, yielding to pMI2. The plasmid was then transformed into the wild type (BD824) strain. For localization studies, strains coexpressing the GFP-tagged *fluG* under native or inducible promoter and the histone H1 (*hhoA*) fused C-terminally to mRFP (mRFP; red fluorescent protein) and mCherry (mCh; cherry red) as nuclear markers, respectively, were constructed (Markina-Iñarrairaegui et al., 2011).

The homologous recombination of the constructs was confirmed by Southern blots. This experiment, using 5′- and 3′-UTR regions as probes, was done as described previously (Garzia et al., 2009). All the mutant strains were sequenced to prove there were no undesired mutations.

### Spore and Dry Cell Mass Measurements

The procedure of obtaining spore and dry cell mass (DCM) ratios was as follows: conidia counts (millions/cm^2^) were obtained after culturing strains in MMA and CMA at 37°C for 72 h. Colony areas were measured using Digimizer Version 4.6.1 (MedCalc Software bvba). Spores were collected by excising the entire colony, soaking the agar in Tween 0.02% (diluted from Tween 20), vortexing twice for 1 min and quantified using a hemocytometer. DCM (mg/cm^2^) from each strain was obtained by growing colonies on a Spectra/Por^®^ 6 Dialysis Membrane with a 3.5 kDa molecular weight cut off (Spectrum Laboratories) and culturing at 37°C for 72 h. The mycelium was then removed from the membrane and dried at 80°C overnight.

The values obtained with the WT and the *ΔfluG* strains were used as references to analyze mutant strains.

### Statistical Analysis of the Results

Quantitative results were obtained as the mean with standard deviation of at least three independent counts, each consisting of three or four biological replicates per strain and condition. The results were subjected to the Grubbs’ test (Grubbs, 1969) using the R software version 3.4.1 (R Core Team, 2008) in order to detect and discard outliers with a confidence level of 95%. A PERMANOVA pair-wise test using an Euclidean distance measurement (*P*-value < 0.05) was performed on the remaining results to justify the significance of differences between strains using PRIMER-E software v6 (Clarke and Gorley, 2006).

### Bioinformatics

The *fluG* DNA sequence (AN4819.2) was retrieved from Ensembl Fungi (Kersey et al., 2018) and homologs in the Aspergilli were identified by Fungal Compara (in Ensembl Fungi). Multiple sequence alignments were performed using the Clustal Omega application provided by EBI (Li et al., 2015). Alignments visualization and analysis were performed with Jalview version 2.10.3b1 (Waterhouse et al., 2009) or GeneDoc version 2.6.002 (Nicholas et al., 1997). FluG protein residue conservation scores among all FluG homologs in the Aspergilli were obtained using the Jensen–Shannon divergence (Capra and Singh, 2007). Identity among all FluG proteins was obtained as an average value of all FluG sequence identities in the Aspergilli compared with the *Aspergillus nidulans* FluG.

Bioinformatic investigations of the FluG sequence were made using the following predictors: SignalIP 4.1 (Nielsen, 2017) for signal-peptide prediction; NLStradamus (Nguyen Ba et al., 2009) and NetNES 1.1 (la Cour et al., 2004) for nuclear localization and export signals prediction, respectively; TMHMM server v2 (Sonnhammer et al., 1998) for prediction of transmembrane helices in protein; epestfind (Rechsteiner and Rogers, 1996) to find PEST motifs as potential proteolytic cleavage sites.

Crystal structure templates for each region were identified using SWISS-MODEL (Biasini et al., 2014), and those with high QMEAN values were further analyzed (Benkert et al., 2009). The highest scoring structures in terms of sequence identity and coverage were used as templates for FluG structural modeling. Protein structures were obtained from the Protein Data Bank (PDB) (Berman et al., 2002). Prior to modeling, the X-ray data was preprocessed and curated (e.g., removal of water, addition of explicit hydrogens) using the Protein Preparation Wizard from the Schrödinger Suite, version 2017-1 (Sastry et al., 2013). Missing side chains were generated using Prime (Jacobson et al., 2004) while the protonation states of each side chain were generated using EPIK at pH = 7 (Shelley et al., 2007). Protein minimization was performed using the OPLS3 force field (Shivakumar et al., 2010; Harder et al., 2016). Homology models were built using program Prime based on the curated reference crystal structure and applying the SWISS-MODEL alignment for each region. The structural and spatial distribution consistency of the FluG site-directed mutant models was validated overlapping these models against the wild type FluG model, using Quick Align and Superimpose tools.

### RNA Isolation

RNA was isolated from the mycelium of strains cultivated in a flask for 18 h at 37°C. Mycelia was collected by filtration through Miracloth (Calbiochem^®^), squeezed to dry and frozen in liquid nitrogen. Total RNA was isolated using the NucleoSpin^®^ RNA Kit (Macherey-Nagel GmbH & Co. KG) and RNA concentration and purity were calculated using a NanoDrop^TM^ 2000c Spectrophotometer (Thermo Fischer Scientific, Waltham, MA, United States). All samples were verified by electrophoresis using a 1.2% (w/v) agarose gel.

### Quantitative RT-PCR

The oligonucleotides employed for quantitative RT-PCR (qPCR) experiments are detailed in Supplementary Table 2. Briefly, cDNA from each investigated sample was synthesized using PrimeScript^TM^ RT reagent Kit (Perfect Real Time) (Takara Bio USA Inc.) from 500 ng of total RNA, following manufacturer’s instructions. The final 20 μL PCR reaction included 1.5 μL of 1:2 diluted cDNA as template, 4 μL of 5x PyroTaq EvaGreen^®^ qPCR Mix Plus with ROX (Cultek Molecular Bioline, Madrid, Spain), and transcript-specific forward and reverse oligonucleotides at a 0.5 μM final concentration. Real time PCR was carried out in a 7500 Real-Time PCR System (Applied Biosystems^®^) according to the manufacturer’s directions. The reaction consisted of 15 min at 95°C of initial denaturation, and 40 cycles of DNA amplification (15 s at 95°C of denaturation, 15 s at 60°C of annealing and 30 s at 72°C of elongation). After each PCR, we performed a melting curve analysis to confirm the specific amplification of a single DNA segments and the absence of non-specific amplified DNA.

The fluorescent signal obtained for each gene was normalized to that obtained with the β-tubulin gene (*benA*) to correct for sampling errors.

### Protein Isolation and Western Blot

Protein extraction from lyophilized samples was performed by the alkaline-lysis extraction, using lysis buffer (0.2 M NaOH, 0.2% β-mercaptoethanol), as described by Hervás-Aguilar and Peñalva (2010).

Tagged protein expression was analyzed by Western blotting. Proteins were resolved in 8% SDS-polyacrylamide gels and electrotransferred to Immun-Blot^®^ PVDF membranes by Trans-Blot^®^ Turbo^TM^ Transfer System (Bio-Rad). Once transference was completed, Ponceau staining was used as validation for loading control. GFP–FluG was detected using mouse anti-GFP (1/5,000; Roche). Peroxidase-conjugated goat anti-mouse IgG immunoglobulin (1/4,000; Jackson ImmunoResearch Lab) cocktail was used as secondary antibody. Peroxidase activity was detected using Clarity^TM^ Western ECL Substrate (Bio-Rad). Chemiluminescence was observed using a ChemiDoc^TM^ XRS+ System (Bio-Rad) and signal intensity was measured with Image Lab^TM^ version 5.2 software (Bio-Rad).

### Fluorescence Microscopy

Conidia of strain BD1181 were cultured in uncoated glass-bottom dishes (Ibidi^®^ GmbH, Germany; 2.5 mL of medium per well) for 16 h at 25°C (Peñalva, 2005). After this period, the medium was replaced with fresh medium supplemented with 100 mM L-threonine (Sigma) in place of D-glucose to induce *alcA(p)::GFP::fluG* expression.

Fluorescence images were acquired using a Zeiss Axio Observer Z1 inverted microscope equipped with a 63x Plan Apochromat 1.4 oil immersion Lens, Axiocam MRm Rev.3 camera, a Zeiss HXP 120C external light source for epifluorescence excitation and fitted with filter set 38HE for green fluorescence (Ex BP 470/40; FT 495; Em BP 525/50) and filter set 43HE for red fluorescence (Ex BP 545/25; FT 570; Em BP 605/70). Numerous samples were observed before taking representative images. Fluorescence levels were measured using Fiji software (Schindelin et al., 2012).

## Results

### DHO-Induced Asexual Development Is Independent of the FluG (UDA) Pathway

**FIGURE 1:**
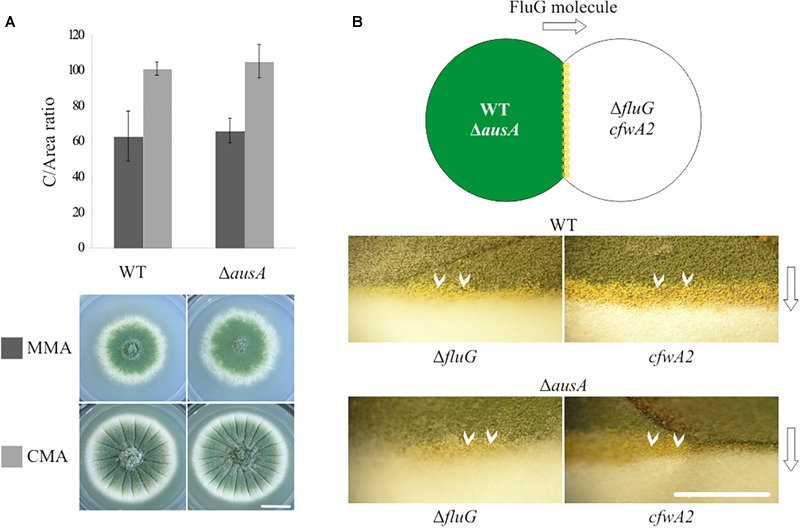
**(A)** Phenotypes and C/area (millions/cm^2^) ratios of the wild type (WT; TN02A3) (reference value) and *ausA* null mutant (*ΔausA*; BD688) in MMA and CMA. Scale bar = 1 cm. **(B)** Phenotypic complementation of a WT and *ΔausA* mutant in contact with a *fluG* null (*ΔfluG*; TTA127.4) and a *cfwA2* (CRO1) mutant. White arrows (∨) indicate the contact area where yellow conidia are visible. Scale bar = 5 mm.

In order to verify previous results by observation that an *ausA* null mutant strain is unaffected in asexual development (Nielsen et al., 2011), an *ΔausA* (*veA1*; BD688) strain was generated and phenotypically characterized in comparison to a parent wild type (WT, *veA1*; TN02A3) strain, as shown in Figure 1A. The values for C and DCM for each strain are provided in Supplementary Table 4. In MMA and CMA, the *C*-values recorded for both WT and *ΔausA* mutant strains were statistically similar. These results indicated that asexual development was not significantly affected in the *ΔausA* mutant.

In addition, a phenotypic complementation experiment confirmed that both WT (*veA1*) and *ΔausA* (*veA1*) strains were able to efficiently complement the sporulation defects of a *ΔfluG* (*veA1*; TTA127.4) and *cfwA2* (*veA1*; CRO1) mutant, as can be observed in Figure 1B. Thus, regardless of the induction effect caused by DHO on asexual development, it cannot be attributed as part of the FluG-dependent UDA pathway. This led to the conclusion that an in-depth study of the FluG protein was required to gain insight on its function.

### The FluG Sequence Codes for a Putative Bifunctional Enzyme

**FIGURE 2:**
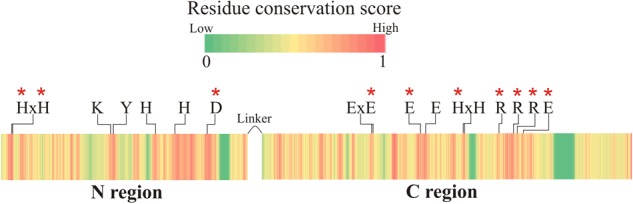
Protein sequence conservation score among all FluG homologs in the Aspergilli. The catalytic residues for both regions are labeled (residue numbers are listed in Tables 2, 3). These residues are conserved in all *Aspergillus* species, and they are all located in highly conserved areas. Red asterisks mark the residues that were mutated.

A protein BLAST of FluG homologs revealed that they were present in all 14 sequenced species of the Aspergilli (Figure 2), with a considerable degree of identity (70 ± 4%) (Supplementary Table 3). Earlier sequence analyses referred to in the introduction (Lee and Adams, 1994; Mathis et al., 1999) had indicated that the *fluG* sequence contained two regions which coded for two putative prokaryotic enzymes. We therefore proceeded to undertake a protein sequence and structure analysis using current applications and databases. A search of available crystal structures using the sequence of the FluG protein showed that the N-terminal region bears similarity with a range of amidohydrolases. The highest sequence coverage and structural homology score was obtained with a putative metal-dependent hydrolase of the TIM-barrel fold (pdb entry 2QPX, coded by gene LSEI_0440) present in *Lactobacillus paracasei* ATCC 334. The 2QPX crystal structure corresponded to the 4–406 amino acid region of FluG, with an identity of 25% and a similarity of 45%. Conjointly, the C-terminal region exhibited the highest scores with a GSI (pdb entry 4LNN, coded by gene *glnA*) from *Bacillus subtilis* (strain 168) (Murray et al., 2013) and a GGL (pdb entry 4HPP, coded by gene PA5508) from *Pseudomonas aeruginosa* PAO1 (Ladner et al., 2012). The 4LNN crystal structure coincided with the 429–865 amino acid region of FluG with a sequence identity of 24% and a similarity of 38%, whereas the 4HPP crystal structure covered the 443–865 amino acid region of FluG, with sequence identity of 22% and a similarity of 33% (Table 1).

**Table 1:** Crystal structure homologs to the N- and C-terminal region.

	N-terminal region	C-terminal region	
PDB entry	2QPX	4LNN	4HPP
Coverage (aa)	4–406	429–865	443–865
Seq. identity (%)	25	24	22
Seq. similarity (%)	43	38	33

*The coverage is shown using the amino acid numbers of FluG. Sequence identity and amino acid positive matches were calculated by Maestro (Schrödinger Suite).*

In order to uncover new features of the protein, we conducted additional studies. Sequence analyses performed using different predictors indicated that the sequence contained neither signal peptides, nuclear localization or export signals nor putative transmembrane domains. However, the PEST predictor marked two potential PEST domains in the C-terminal region (aa571 to aa590 with a score of 14.62 and aa529 to aa541 with a score of 6.84, respectively). Analyses of the genomic DNA and mRNA of *fluG* sequence revealed two predicted introns between nucleotides 1126–1176, and 2420–2472. A previous RNA-seq study (Garzia et al., 2013) reported a single mRNA transcript of 2,598 nucleotides, in which both introns are spliced out.

### Localization of FluG *in vivo*

**FIGURE 3:**
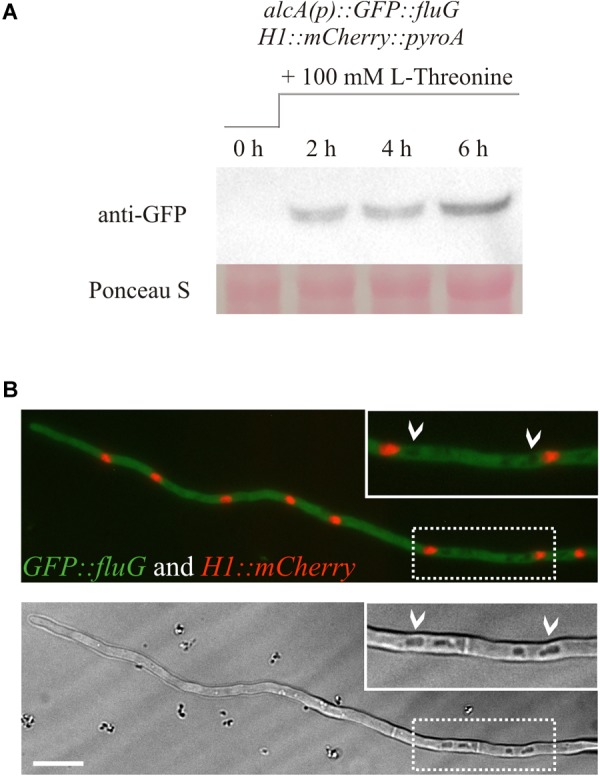
**(A)** Western blot showing the protein expression of the N-terminally GFP-tagged FluG construct under the control of the *alcA* promoter. Induction of the *alcA(p)::GFP::fluG* strain shows an increment on the expression of GFP–FluG in time (2, 4, and 6 h). **(B)** Images obtained in the fluorescence microscopy show that FluG presents principally a cytoplasmic localization, excluded from the nuclei and is not detectable in vacuoles (white arrows). Scale bar = 10 μm.

A GFP-tagged version of the protein was used for *in vivo* localization. Strains tagged in the N- and C- terminal regions of the protein were grown in MMA and CMA for 72 h, and their phenotype observed (Supplementary Figure 1). The strain tagged in the C-terminal presented a fluffy phenotype, indicative of loss of function, while the N-terminally tagged strain presented a wild type phenotype. Hence, the latter was chosen to perform localization experiments. In order to determine the incubation time at which the protein was sufficiently expressed for observation, the pattern of expression in liquid vegetative culture was monitored by Western blot at time intervals. In the case that *fluG* expression was controlled under the *alcA* promoter, a shift to a medium containing threonine was conducted at time zero. The results revealed that GFP::FluG protein could only be detected in the strain *alcA(p)::GFP::fluG* 2 h after medium shift to threonine containing medium (Figure 3A).

Consistent with the results obtained, no GFP–FluG fluorescence was detected in the strain bearing the native *fluG* promoter (not shown). In the strain with the *alcA* inducible promoter, GFP–FluG was localized 2 h after the medium shift in the cytoplasm throughout the length of the hypha. Nuclei were stained by tagging histone H1 tagged with mCherry fluorescence, but no GFP signal was detected in the nuclear compartment as shown in Figure 3B. In addition, the protein appeared to be absent from the lumen of vacuoles (white arrows). Taken together, our microscopic observations indicated that FluG was evenly distributed throughout the cytoplasm of vegetative cells.

### Computational Analysis of the N- and C-Terminal Regions

**FIGURE 4:**
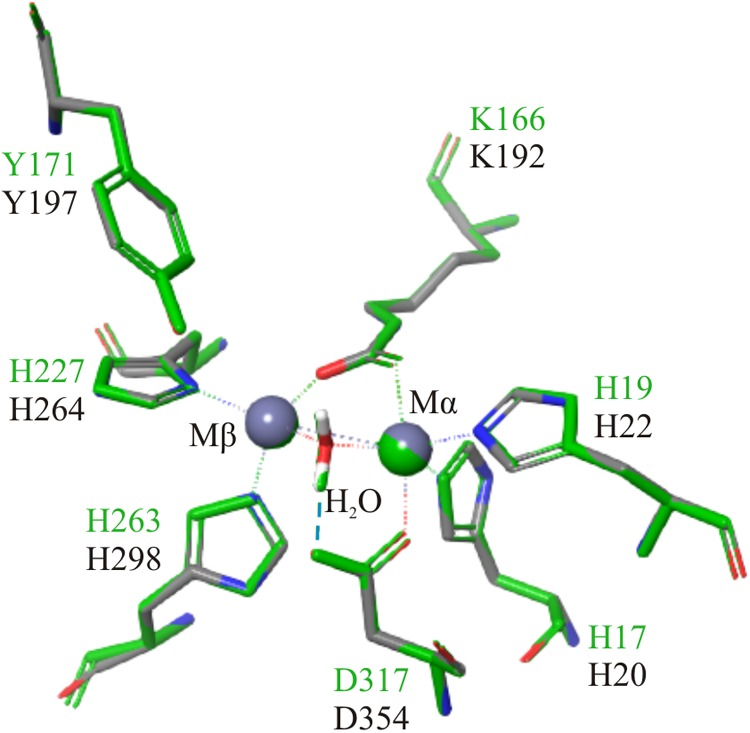
Structural alignment of 2QPX (green) refined model, with the FluG modeled structure (gray), based on 2QPX template. The Mα is coordinated to H20, H22, K192, and D354; the Mβ is coordinated to K192, H264, and H298. A water molecule is coordinated to both metals and D354 (blue dashed line).

The closest homolog to the N-terminal region of FluG, 2QPX, was used as a template to model structural features. The residues shared by both sequences and their predicted functions are shown in Table 2. In 2QPX, residues H17, H19 and D317 coordinate to the first Zn^2+^ (Mα), whilst H227 and H263 coordinate to the second Zn^2+^ (Mβ). In addition, a lysine (K166) undergoes a post-translational carbamylation to bridge both Zn^2+^ ions, and a tyrosine (Y171) is predicted to interact with the substrate in certain amidohydrolases. The N-terminal region of FluG conserves all the above-mentioned residues (H20, H22, K192, H264, H298, and D354), along with an appropriate spatial arrangement to bind and bridge both metals, together with the tyrosine (Y197) that could interact with the substrate (Figure 4). These residues are conserved through all the Aspergilli FluG homologs, as marked in Figure 2.

**Table 2:** Predicted catalytic residues in 2QPX, and their FluG equivalents.

Residues		Predicted role
2QPX	FluG	
H17	H20	Coordinates with the Mα metal
H19	H22	Coordinates with the Mα metal
K166	K192	Once carbamylated (KCX), bridges both Mα and Mβ
Y171	Y197	Coordinates with the substrate to aid the cleavage of the bond
H227	H264	Coordinates with Mβ
H263	H298	Coordinates with Mβ
D317	D354	Coordinates with Mα

*Metals labeled as Mα and Mβ correspond to the first and the second Zn^*2+*^ atoms. The lysine residue (K166) undergoes a post translational modification that results in a carbamylation.*

**FIGURE 5:**
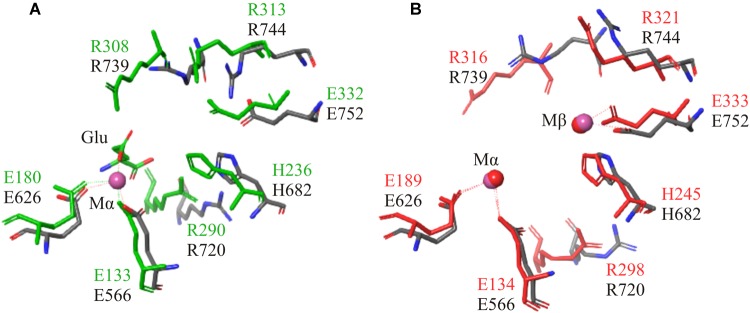
Structural superposition of both GGL (4HPP) and GSI (4LNN) with the FluG models. Only mutated residues from this work are displayed. **(A)** 4HPP refined template (green) with the FluG modeled structure (gray), based on 4HPP. Coordination of the residues between the single Mg^2+^ ion (Mα interacts with residues E566 and E626) and glutamate ligand (Glu interacts with residue R720) in 4HPP is considerably similar in FluG. **(B)** 4LNN refined template (red) with the FluG modeled structure (gray), based on 4LNN. Coordination between both Mg^2+^ ions (Mα interacts with residues E566 and E626; Mβ interacts with H682 and E752) and residues in FluG shows significant similarity to 4LNN.

Structural alignment of the C-terminal region using the crystal structures of a GSI (4LNN) and a GGL (4HPP), also showed homology in key catalytic residues (Table 3). The amino acids that coordinate to the metal ions in both GGL (one Mg^2+^ [Mα] coordinated to E133, E180, and E187) (Figure 5A) and GSI (two Mg^2+^; Mα coordinated to E134, E189 and E196, and Mβ coordinated to E132, H245, and E333) (Figure 5B) were structurally conserved in the C-terminal region of FluG (E566, E626, E633, H682, and E752). Moreover, the residues that bind to the glutamate ligand (R290 in 4HPP; R298 in 4LNN) and interact with ATP (H238, R308, and R313 in 4HPP; N247, R316, and R321 in 4LNN) were present in FluG (R720 for the glutamate; H682, R739, and R744 for the ATP, respectively). The residues listed in Table 3 are fully conserved in FluG for all the Aspergilli (Figure 2).

**Table 3:** Predicted role of the catalytic residues from 4LNN (GSI), 4HPP (GGL), and FluG.

Residues			Predicted role
4LNN	4HPP	FluG	
D53	V42	S481	Removes a proton from the ammonium ion to create ammonia
E132	E131	E564	Binds Mβ
E134	E133	E566	Binds Mα; susceptible to interact with inhibitors
E189	E180	E626	Binds Mα and the NH_3_ molecule
E196	E187	E633	Binds Mα
H245	H236	H682	Binds Mβ, via pros nitrogen
N247	H238	H684	Interacts with ATP γ-phosphate
R298	R290	R720	Interacts with the amide group of the glutamate
E304	W296	W726	Abstracts a proton from the transition state to form glutamine and ADP
R316	R308	R739	Polarizes the bound ATP γ-phosphate that forms the γ-glutamyl phosphate
R321	R313	R744	Contacts with the ATP in the transition state
E333	E332	E752	Binds Mβ

*Metals labeled as Mα and Mβ correspond to the first and the second Mg^*2+*^ atoms. Mα and Mβ are located in the first and the second catalytic domain, respectively.*

On the other hand, the GSI crystal structure (4LNN) contains a glutamate residue (E304) that abstracts a proton from the transition state to form glutamine and ADP. It is worth noting, however, that the GGL crystal structure and the FluG (C-terminal) model each contain a tryptophan residue in the same position (W296 and W726, respectively), which cannot fulfill the function of glutamate. Furthermore, the conserved residue D53 in GSI that removes a proton from the ammonium ion to create ammonia is aligned with V42 and S481 in GGL and FluG, respectively; neither of the aforementioned amino acids can perform the role of a GSI.

An important aspect that differentiates GGL and GSI enzymes is the fact GGL forms hexameric rings and GSI enzymes form two hexameric subunits that assemble into a dodecameric unit. The formation of the dodecamer depends on the conformation that the C-terminal end of these enzymes adopts. Structural alignments performed with SWISSMODEL using 4LNN and 4HPP crystals revealed that the C-terminal end of FluG exhibited higher QMEAN values with the 4HPP crystal in this region (not shown).

### Phenotypic Characterization of WT and *ΔfluG* Mutant

**FIGURE 6:**
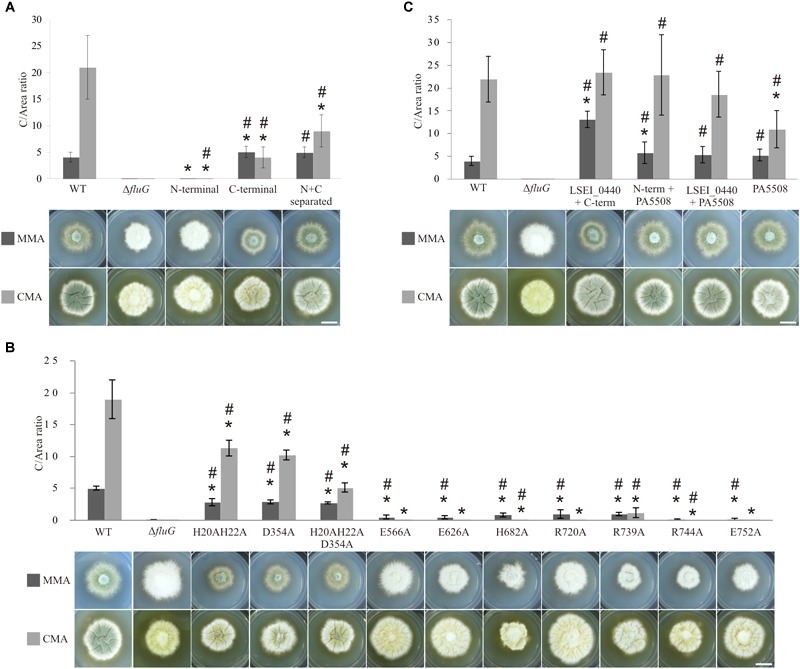
Phenotypes and C/area (millions/cm^2^) ratios of all the FluG mutants. **(A)** Wild type (WT) along with *fluG* null (*ΔfluG*), N-terminal (aa1–aa406), C-terminal (aa427–aa865) regions and N- and C-terminal separated regions (N+C separated) mutants in MMA and CMA. **(B)** FluG point-mutations in both regions. **(C)** Heterologous substitutions, showing LSEI_0440+C-term (FluG), N-term (FluG)+PA5508, LSEI_0440+PA5508 and PA5508 only. Note that different scales were used in each panel to better represent the differences in C/area (millions/cm^2^) values. The *C*-values that are statistically different (*P*-value < 0.05) to the WT (^∗^) and the *ΔfluG* (#) are marked. Scale bar = 1 cm.

The WT and *ΔfluG* strains were established as references for comparisons with strains containing modifications, and their phenotypes were characterized in minimal (MMA) and complete medium (CMA). The conidial production in millions per centimeter squared of colony surface (C) in 72 h old colonies of both strains is presented in Figure 6A. Numerical values for C and DCM are separately provided in Supplementary Table 5. The WT colonies presented a *C*-value of 4.1 in MMA, distinctly greater than the *ΔfluG* strain, 0.02, but significantly lower DCM values. In CMA, the WT strain presented increased C and DCM indices with respect to MMA. The *ΔfluG* strain showed even lower conidial counts than in MMA and increased DCM values. Taken together, the results indicate that, under nutrient limitation (MMA), the wild type strain distributes resources between conidiation and growth, while the *ΔfluG* mutant destines resources exclusively to growth. However, under nutrient sufficiency (CMA), both strains attained apparently maximum growth levels, and accentuated their differences in conidial production. It is due to these clearly discernible differences, that the *C*-value was considered as the principal phenotypic indicator of functionality when examining genetically modified strains.

### The N- and C-Terminal Regions of FluG Perform Distinct Functions

Each of the two FluG regions was expressed separately under the control of the native promoter. The results shown in Figure 6A indicate that in MMA, the mutant expressing only the C-terminal region presented a *C*-value significantly greater to that of the WT strain, while in CMA, it was one-fifth of the WT *C*-value. These results indicate that the C-terminal region was capable of fulfilling the role of the complete FluG polypeptide, but the absence of the N-terminal region limited its efficacy in CMA.

The strain exclusively expressing the N-terminal region, presented a *C*-value which was significantly similar to that of the *ΔfluG* mutant in MMA, but no so in CMA media. These results support the interpretation that the C-terminal region performs the core function of the protein in asexual development, but that the N-terminal region may provide a supporting function under conditions of high nutrient availability.

A strain expressing both N- and C-terminal regions as two separate polypeptides, each under the control of the native promoter of *fluG* was analyzed (N+C separated) (Figure 6A). The recorded *C*-value in MMA was significantly similar to that recorded for the WT and C-terminal region strains. In CMA, the value was at an intermediate point between both strains (Supplementary Table 5). These results not only indicate that both regions are functional when separately expressed, but support the view that the N-terminal region performs a relevant supportive role in conidial production, especially when both regions are expressed in the same polypeptide and under nutrient rich conditions.

In order to ascertain that the phenotypes observed were not affected by altered gene expression levels, strains were examined by qPCR and compared to that of the WT type strain, as shown in Supplementary Figure 2. With the exception of the *ΔfluG* mutant, all mutant strains presented transcript levels equal or higher than those observed in the WT strain.

### Targeted Amino Acid Substitutions Affect Biological Function

Previously reported mutations on residues that were homologous to H20, H22, and D354 in the N-terminal region of FluG had been shown to be essential for amidohydrolase activity (Kim and Kim, 1998; Li et al., 2006; Lohkamp et al., 2006; Nguyen et al., 2009). Therefore, double (H20–H22), single (D354), and triple (H20–H22–D354) alanine substitution mutants were constructed. A similar strategy was followed for the C-terminal region, where residues E566, E626, H682, R720, R739, R744, and E752 were chosen based on previous reports (Kurihara et al., 2008; Ladner et al., 2012; Murray et al., 2013). The targeted residues are marked with asterisks in Figure 2. All these amino acid substitutions tested negative for propensity to disrupt structure using homology modeling (see “Materials and Methods” section). The observed phenotypes for the full set of mutants are shown in Figure 6B.

All the strains carrying mutations in the N-terminal region exhibited significantly lower *C*-values with respect to the WT reference in both media. In MMA, the *C*-values obtained for mutants H20–H22, D354, and H20–H22–D354 were significantly similar. In CMA, however, significant differences between the H20–H22, D354, and H20–H22–D354 mutants were observed with the triple mutant showing the maximum reduction in *C*-value (Figure 6B). This indicates an additive effect of the mutations, only under high nutrient availability.

In the C-terminal region, residues E566, E626, H682, R720, R739, R744, and E752 were separately substituted by alanine. All the mutant strains presented statistically lower *C*-values in MMA when compared to the WT. These low values were nevertheless significantly higher than those obtained in the *ΔfluG* strain, indicating that single amino acid modifications resulted in a partial inactivation of the activity. In CMA, the *C*-values were negligible for the majority of mutants in comparison to the WT strain. When compared to the *ΔfluG* strain, the majority of the mutants were statistically similar. Three mutants however (H682A, R739A, and R744A) were statistically different, indicating a partial inactivation (Figure 6B). Strain R739A, presented a diverging phenotype yielding statistically higher *C*-values in CMA when compared to the other mutants of this region.

The quantification of *fluG* expression in all the tested mutants indicated that the transcript was at least as abundant as in the WT strain (Supplementary Figure 2).

Taken together, the results obtained by these substitutions support the view that residues predicted to perform catalytic activity in both N- and C-terminal regions are involved in the biological functions earlier recognized for their corresponding regions.

### Heterologous Expression of N- and C-Terminal Region Homologs

Given the sequence and structural homologies observed between both N- and C-terminal regions with those of *Lactobacillus paracasei* (LSEI_0440) and *Pseudomonas aeruginosa* (PA5508), we constructed strains in which the exogenous sequences were expressed under the control of the *fluG* promoter in combinations that included three protein chimeras (LSEI_0440+C-terminal; N-terminal+PA5508; LSEI_0440+PA5508) and one single polypeptide (PA5508).

The phenotypes of the mutant strains are shown in Figure 6C. The *C*-values obtained in the LSEI_0440+C-terminal and N-terminal+PA5508 strains were significantly higher than the WT strain in MMA. In the same conditions, the PA5508 and LSEI_0440+PA5508 exhibited statistically similar *C*-values when compared to the WT strain. On the other hand, the *C*-values of the three protein chimeras (LSEI_0440+C-terminal; N-terminal+PA5508; LSEI_0440+PA5508) were statistically similar to the WT reference in CMA. However, the strain expressing the PA5508 polypeptide alone exhibited a significantly lower value compared to the WT in the same medium.

The results clearly show that the protein encoded by gene LSEI_0440 could functionally replace the N-terminal region of FluG; similarly, the protein encoded by PA5508 fulfilled the role of the C-terminal region of FluG, either as a single polypeptide (PA5508 mutant) or fused to the N-terminal region of FluG (N-terminal+PA5508 chimera). In line with the aforementioned results, the fact that the chimera expressing a fusion protein of LSEI_440 and PA5508 exhibits a statistically comparable phenotype to a WT strain confirms that both bacterial enzymes can work jointly in replacing both FluG regions.

## Discussion

This investigation initially attempted to clarify the role of DHO as a conidiation-inducing secondary metabolite in relation to FluG function. Previous evidence that secondary metabolites (such as DHO) may play a role in the induction of sporulation (Márquez-Fernández et al., 2007; Rodriguez-Urra et al., 2012), was in conflict with observations describing a null *ausA* mutant, unable to synthesize DHO, as conidial (Nielsen et al., 2011). This investigation moreover showed that such mutants can produce and transmit the conidiation-inducing factor to an adjacent *ΔfluG* colony. Thus, DHO-induced conidiation is not functionally related to the FluG pathway. This scenario led us to conduct an updated analysis of the FluG sequence to obtain a molecular outlook on its function.

Preliminary bioinformatic approaches indicated that FluG comprises two putative catalytic units, connected in the same polypeptide by a linker region, which likely function as a bifunctional enzyme performing two independent reactions. *In vivo* fluorescence microscopy experiments confirmed that FluG is localized throughout the cytoplasm, indicating that the protein may participate in a metabolic process that is ubiquitous to the whole mycelium. However, considerable evidence shows that fungal mycelia are spatially and functionally structured. For example, in gene expression (Levin et al., 2007; Vinck et al., 2011) and protein secretion (Krijgsheld et al., 2012). In this regard, the role of FluG might be hypothesized as the mycelium-wide biosynthesis of a metabolic product or signal specifically required for conidiophore biogenesis. Conidiophores are produced at specific points in the network, likely acting as metabolic sinks. This is in accordance with reported cytosolic streaming directed at the conidiophore (Bleichrodt et al., 2013). Thus, further molecular studies involving FluG function should take the mycelial context into consideration.

The C-terminal region was confirmed as necessary and sufficient for the induction of development and presented clear sequence similarities with both GSI and GGL enzyme sequences. Close examination of the differences between these two types of enzymes revealed that FluG more closely matches the latter (Kurihara et al., 2008; Ladner et al., 2012; Takeo et al., 2013). To address this point, a site-directed mutational approach was planned to determine the importance of the predicted catalytic residues present in GSI and GGL. However, GSI catalytic activity involves the interaction between two residues (a glutamate and an aspartate) that shield the γ-glutamyl phosphate intermediate and deprotonate ammonium (forming ammonia) to attack the γ-glutamyl phosphate (Murray et al., 2013). Interestingly, these key acidic residues are not conserved in the sequences of GGL, FluG or those GSI homologs that reportedly do not catalyze the synthesis of glutamine, but γ-glutamylation reactions (Kurihara et al., 2008; Ladner et al., 2012; Takeo et al., 2013; Krysenko et al., 2017). On the other hand, residues acting on metal binding and glutamate orientation are usually conserved among these enzymes (Ladner et al., 2012), as was confirmed in FluG. The attachment of glutamate has been attributed to exert a scaffolding or protective role on substrates destined to specific metabolic fates (Walker and van der Donk, 2016), as will be discussed below. The strain in which the GGL coded by PA5508 replaced the C-terminal region of FluG recovered the functionality of the WT strain in all tested mutants and conditions. Earlier reports associated this gene with the polyamine utilization pathway along with six other paralog genes in *Pseudomonas aeruginosa* PAO1. Surprisingly, PA5508 was the only gene that was not induced by polyamine substrates (Yao et al., 2011). Later investigations performed by Ladner et al. (2012) showed that the enzymatic substrate preference of PA5508 was stronger for bulky amines (catecholamines) rather than linear amines (polyamines). Whether the substrate employed by the FluG C-terminal region bares any resemblance to the ones elucidated by Ladner et al. (2012) is a question that is being actively followed up.

Another notable difference between GGL and GSI enzymes is that GGL forms single hexameric rings, whilst GSI enzymes form two hexameric rings which then arrange to form a dodecameric unit. This assembly occurs via the C-terminal helix of the polypeptide, known as the “helical thong,” that extends away from the rest of the subunit and interacts with the second hexameric ring. In GGL, however, the C-terminal helix is curved toward the protein, preventing it from interacting with another ring (Ladner et al., 2012). Our structural alignments revealed that the FluG C-terminal end exhibited higher structural similarities with the GGL, thus suggesting that it might also adopt a similar “helical thong” conformation. Furthermore, this evidence might also explain the loss of function observed in this study when tagging FluG at the C-terminus.

The putative amidohydrolase sequence contained in the N-terminal region of FluG is a member of a well-studied superfamily of enzymes that share common structural features. The vast majority of the amidohydrolase crystal structures display a characteristic TIM-barrel fold consisting of eight parallel β-strands connected by eight α-helices. Furthermore, they share conserved metal ligands and catalytically important residues, that bind one or two metal ions acting as catalytic sites, together with four histidines and one aspartate acting as catalytic bonds (Seibert and Raushel, 2005). These motifs are all present in the N-terminal region of FluG, coupled with the structural conservation of a lysine and a tyrosine that could bridge both metals and bind to the substrate, respectively (Hsieh et al., 2013). The phenotypic analysis of the H20A–H22A double mutant and the single D354A mutant showed partial inactivation, and the triple H20A–H22A–D354A mutant displayed greater level of functional inactivation suggesting an additive effect. This result is consistent with the reported importance that these residues have in the coordination of the Mα; the process is followed by the lysine carbamylation, a necessary step to bind the Mβ (Hsieh et al., 2013). Moreover, the phenotype shown by the triple mutant resembled the one observed in the mutant lacking the whole region.

Although details of the reaction purportedly catalyzed by the N-terminal region of FluG remain unresolved at this point, the predicted structural features point to the hydrolysis of a bond, excluding deaminase (elimination of ammonia from aromatic bases) activity. Alignments based exclusively on proteins with solved crystal structures, showed a notable residue conservation between the N-terminal region of FluG and LSEI_0440. Furthermore, both enzymes exhibited strong similarities with cyclic amidohydrolases (Nam et al., 2005), such as dihydropyrimidinases (Lohkamp et al., 2006; Hsieh et al., 2013) and hydantoinases (Cheon et al., 2002) that take part in the pyrimidine degradation pathway. Unfortunately, although LSEI_0440 could functionally replace the amidohydrolase region of FluG, the actual activity of this enzyme remains unknown. Previous reports on the occurrence of promiscuous enzymes among the amidohydrolase superfamily (Baier et al., 2016) should be taken into consideration, when evaluating the functional replacement of the N-terminal of FluG by LSEI_0440. Bearing in mind that the amidohydrolase superfamily has emerged from a common ancestor (Seibert and Raushel, 2005) and that ancestral enzymes may have multifunctional (non-specialized) or promiscuous catalytic activity (Baier et al., 2016), LSEI_0440 might as well retain enzymatic characteristics that confer substrate or catalytic promiscuity, compared to a more specialized role of the N-terminal of FluG. On the other hand, the inherent Michaelis constant that LSEI_0440 or the N-terminal of FluG have, might as well differ; considering that the LSEI_0440 substitution leads to a highly sporulating phenotype, it suggests that this enzyme could have a lower K_M_. Conversely, evidence of possible post translational modifications in FluG (Lee and Adams, 1994) could explain the inability of the LSEI_0440+C-terminal strain to maintain wild type *C*-values in the tested conditions. It is likely that the N-terminal of FluG might have a regulatory domain that has not been uncovered by the predictors. Ongoing experiments for the clarification of this aspect of FluG function are currently underway.

The combined results of this investigation indicate that FluG may function as a bifunctional enzyme with each domain sharing high similarities to its’ respective prokaryotic homologs. In prokaryotes, they may or may not participate in a common metabolic pathway, but in Aspergilli, they are joined by a linker region to form a bifunctional enzyme. Supporting this relation, the mutant in which both N- and C-terminal regions were substituted by LSEI_0440 and PA5508, respectively, restored *in vivo* functionality. The occurrence of genes that code for more than one enzyme has been attributed to fusion and fission events driven by paralogous evolution from bacteria to fungi (Mathis et al., 2000) or between different fungal species via chromosomal translocation or chromosomal inversion (Leonard and Richards, 2012). In bacterial gene clusters, close disposition between predicted GSI and amidohydrolase sequences is apparent, possibly indicating a functional connection (Strong et al., 2003; Iyer et al., 2009). However, the phenotypic effect caused by the separation of the N- and C-terminal region confirms that both proteins perform their roles more efficiently as a single polypeptide, than as two separate entities. However, our experimental methods and conditions may not have bridged the specific conditions that optimization provided by the linkage of the two halves of FluG solved over the course of evolution of FluG. Surely, the totality of inputs into developmental decisions of Aspergilli have not been elucidated. The unresolved questions of FluGs specifics leave the door open to new discoveries in this field. In any case, our results consistently support the view that the N-terminal amidohydrolase activity plays a supporting role to the essential C-terminal catalytic activity, especially under nutrient rich conditions.

In functional terms, the N-terminal amidohydrolase activity is not necessary for the induction of asexual development but is required to maintain the *C*-value of the colony under conditions of high nutrient availability. This finding could be explained by a model where the amidohydrolase could utilize an accumulating metabolic intermediate as a substrate that would otherwise be consumed by an alternative pathway, possibly supporting vegetative growth. The product of the amidohydrolase reaction, in turn, could constitute a substrate of the C-terminal region, as an element of a metabolic branch which could feed into a development-related pathway. The hypothetical role of these pathways would be to scavenge nitrogen-rich intermediates through glutamylation in order to feed alternative pathways (Walker and van der Donk, 2016). Furthermore, nitrogen acquisition through glutamylated intermediates in bacteria has been reported to occur in the biosynthesis of polyamines (Kurihara et al., 2008; Krysenko et al., 2017) and certain catecholamines (Ladner et al., 2012; Takeo et al., 2013). Regardless of the predictions obtained for the C-terminal GSI enzymatic nature to date, phenotypic evidence obtained in this investigation sustains that FluG performs the γ-glutamylation reaction of yet unknown compounds, assisted by the N-terminal region. Given this scenario, it would seem that the purported substrate of FluG could undergo at least two metabolic fates, namely the promotion of growth, as observed in mutants affected in C-terminal activity, or asexual development. The intracellular pool of that substrate may be much greater under high nutrient availability and scavenging by the N-terminal amidohydrolase activity may be an important factor in balancing the ratio of growth and asexual development within the colony.

## Author Contributions

MI-S conducted and designed the experimental work, analyzed the data, and wrote the manuscript. LT-G conducted and designed the experimental work, carried out the statistical treatment of the results, and revised the manuscript. MC contributed to the bioinformatic approach, validated the final predictions, and revised the manuscript. UU co-conceived the work, ensured the scientific issue was appropriately investigated, ensured the integrity of the work, contributed to writing the manuscript, and revised and approved the final version for submission.

## Conflict of Interest Statement

The authors declare that the research was conducted in the absence of any commercial or financial relationships that could be construed as a potential conflict of interest.

## References

[B1] Adams T. H. Wieser J. K. Yu J. H. (1998). Asexual sporulation in *Aspergillus nidulans*. *Microbiol. Mol. Biol. Rev.* 62 35–54. 9529886 10.1128/mmbr.62.1.35-54.1998 PMC98905

[B2] Baier F. Copp J. N. Tokuriki N. (2016). Evolution of enzyme superfamilies: comprehensive exploration of sequence-function relationships. *Biochem.* 55 6375–6388. 10.1021/acs.biochem.6b00723 27802036

[B3] Benkert P. Künzli M. Schwede T. (2009). QMEAN server for protein model quality estimation. *Nucleic Acids Res.* 37 W510–W514. 10.1093/nar/gkp322 19429685 PMC2703985

[B4] Berman H. M. Battistuz T. Bhat T. N. Bluhm W. F. Bourne P. E. Burkhardt K. (2002). The protein data bank. *Acta Crystallogr. D Struct. Biol. D* 58 899–907. 10.1107/S0907444902003451 12037327

[B5] Biasini M. Bienert S. Waterhouse A. Arnold K. Studer G. Schmidt T. (2014). SWISS-MODEL: modelling protein tertiary and quaternary structure using evolutionary information. *Nucleic Acids Res.* 42 W252–W258. 10.1093/nar/gku340 24782522 PMC4086089

[B6] Bleichrodt R. Vinck A. Krijgsheld P. van Leeuwen M. R. Dijksterhuis J. Wösten H. A. (2013). Cytosolic streaming in vegetative mycelium and aerial structures of *Aspergillus niger*. *Stud. Mycol.* 74 31–46. 10.3114/sim0007 23450745 PMC3563289

[B7] Capra J. A. Singh M. (2007). Predicting functionally important residues from sequence conservation. *Bioinformatics* 23 1875–1882. 10.1093/bioinformatics/btm270 17519246

[B8] Chang P. K. Scharfenstein L. L. Mack B. Ehrlich K. C. (2012). Deletion of the *Aspergillus flavus* orthologue of *A. nidulans fluG* reduces conidiation and promotes production of sclerotia but does not abolish aflatoxin biosynthesis. *Appl. Environ. Microbiol.* 78 7557–7563. 10.1128/AEM.01241-12 22904054 PMC3485703

[B9] Cheon Y. H. Kim H. S. Han K. H. Abendroth J. Niefind K. Schomburg D. (2002). Crystal structure of D-Hydantoinase from *Bacillus stearothermophilus*: insight into the stereochemistry of enantioselectivity. *Biochemistry* 41 9410–9417. 10.1021/bi0201567 12135362

[B10] Clarke K. R. Gorley R. N. (2006). *PRIMER v6: User Manual/Tutorial*. Plymouth: PRIMER-E.

[B11] Doskočilová A. Plíhal O. Volc J. Chumová J. Kourová H. Halada P. (2011). A nodulin/glutamine synthetase-like fusion protein is implicated in the regulation of root morphogenesis and in signalling triggered by flagellin. *Planta* 234 459–476. 10.1007/s00425-011-1419-7 21533644

[B12] D’Souza C. A. Lee B. N. Adams T. H. (2001). Characterization of the role of the FluG protein in asexual development of *Aspergillus nidulans*. *Genetics* 158 1027–1036. 11454752 10.1093/genetics/158.3.1027 PMC1461723

[B13] Dyer P. S. O’Gorman C. M. (2012). Sexual development and cryptic sexuality in fungi: insights from *Aspergillus* species. *FEMS Microbiol. Rev.* 36 165–192. 10.1111/j.1574-6976.2011.00308.x 22091779

[B14] Emri T. Molnár Z. Pusztahelyi T. Varecza Z. Pócsi I. (2005). The FluG-BrlA pathway contributes to the initialisation of autolysis in submerged *Aspergillus nidulans* cultures. *Mycol. Res.* 109(Pt 7) 757–763. 10.1017/S0953756205003023 16121561

[B15] Garzia A. Etxebeste O. Herrero-Garcia E. Fischer R. Espeso E. A. Ugalde U. (2009). *Aspergillus nidulans* FlbE is an upstream developmental activator of conidiation functionally associated with the putative transcription factor FlbB. *Mol. Microbiol.* 71 172–184. 10.1111/j.1365-2958.2008.06520.x 19007409

[B16] Garzia A. Etxebeste O. Rodriguez-Romero J. Fischer R. Espeso E. A. Ugalde U. (2013). Transcriptional changes in the transition from vegetative cells to asexual development in the model fungus *Aspergillus nidulans*. *Eukaryot. Cell* 12 311–321. 10.1128/EC.00274-12 23264642 PMC3571305

[B17] Grubbs F. E. (1969). Procedures for detecting outlying observations in samples. *Technometrics* 11 1–21. 10.1080/00401706.1969.10490657

[B18] Harder E. Damm W. Maple J. Wu C. Reboul M. Xiang J. Y. (2016). OPLS3: a force field providing broad coverage of drug-like small molecules and proteins. *J. Chem. Theory Comput.* 12 281–296. 10.1021/acs.jctc.5b00864 26584231

[B19] Hervás-Aguilar A. Peñalva M. A. (2010). Endocytic machinery protein SlaB is dispensable for polarity establishment but necessary for polarity maintenance in hyphal tip cells of *Aspergillus nidulans*. *Eukaryot. Cell* 9 1504–1518. 10.1128/EC.00119-10 20693304 PMC2950435

[B20] Hsieh Y. C. Chen M. C. Hsu C. C. Chan S. I. Yang Y. S. Chen C. J. (2013). Crystal structures of vertebrate dihydropyrimidinase and complexes from *Tetraodon nigroviridis* with lysine carbamylation metal and structural requirements for post-translational modification and function. *J. Biol. Chem.* 288 30645–30658. 10.1074/jbc.M113.496778 24005677 PMC3798535

[B21] Iyer L. M. Abhiman S. Maxwell Burroughs A. Aravind L. (2009). Amidoligases with ATP-grasp, glutamine synthetase-like and acetyltransferase-like domains: synthesis of novel metabolites and peptide modifications of proteins. *Mol. Biosyst.* 5 1636–1660. 10.1039/b917682a 20023723 PMC3268129

[B22] Jacobson M. P. Pincus D. L. Rapp C. S. Day T. J. F. Honig B. Shaw D. E. (2004). A hierarchical approach to All-atom protein loop prediction. *Proteins* 55 351–367. 10.1002/prot.10613 15048827

[B23] Käfer E. (1965). Origins of translocations in *Aspergillus nidulans*. *Genetics* 52 217–232. 5857597 10.1093/genetics/52.1.217 PMC1210839

[B24] Kersey P. J. Allen J. E. Allot A. Barba M. Boddu S. Bolt B. J. (2018). Ensembl genomes 2018: an integrated omics infrastructure for non-vertebrate species. *Nucleic Acids Res.* 46 D802–D808. 10.1093/nar/gkx1011 29092050 PMC5753204

[B25] Kim G. J. Kim H. S. (1998). Identification of the structural similarity in the functionally related amidohydrolases acting on the cyclic amide ring. *Biochem. J.* 330(Pt 1) 295–302. 10.1042/bj3300295 9537960 PMC1219176

[B26] Korbie D. J. Mattick J. S. (2008). Touchdown PCR for increased specificity and sensitivity in PCR amplification. *Nat. Protoc.* 3 1452–1456. 10.1038/nprot.2008.133 18772872

[B27] Krijgsheld P. Altelaar A. F. Post H. Ringrose J. H. Müller W. H. Heck A. J. (2012). Spatially resolving the secretome within the mycelium of the cell factory *Aspergillus niger*. *J Proteome Res.* 11 2807–2818. 10.1021/pr201157b 22443316

[B28] Krysenko S. Okoniewski N. Kulik A. Matthews A. Grimpo J. Wohlleben W. (2017). Gamma-glutamylpolyamine synthetase GlnA3 is involved in the first step of polyamine degradation pathway in *Streptomyces coelicolor* M145. *Front. Microbiol.* 8:726. 10.3389/fmicb.2017.00726 28487688 PMC5403932

[B29] Kurihara S. Oda S. Tsuboi Y. Kim H. G. Oshida M. Kumagai H. (2008). γ-Glutamylputrescine synthetase in the putrescine utilization pathway of *Escherichia coli* K-12. *J. Biol. Chem.* 283 19981–19990. 10.1074/jbc.M800133200 18495664

[B30] la Cour T. Kiemer L. Mølgaard A. Gupta R. Skriver K. Brunak S. (2004). Analysis and prediction of leucine-rich nuclear export signals. *Protein Eng. Des. Sel.* 17 527–536. 10.1093/protein/gzh062 15314210

[B31] Ladner J. E. Atanasova V. Dolezelova Z. Parsons J. F. (2012). Structure and activity of PA5508, a hexameric glutamine synthetase homologue. *Biochemistry* 51 10121–10123. 10.1021/bi3014856 23234431

[B32] Lee B. N. Adams T. H. (1994). The *Aspergillus nidulans fluG* gene is required for production of an extracellular developmental signal and is related to prokaryotic glutamine synthetase I. *Genes Dev.* 8 641–651. 10.1101/gad.8.6.641 7926755

[B33] Leonard G. Richards T. A. (2012). Genome-scale comparative analysis of gene fusions, gene fissions, and the fungal tree of life. *Proc. Natl. Acad. Sci. U.S.A.* 109 21402–21407. 10.1073/pnas.1210909110 23236161 PMC3535628

[B34] Levin A. M. de Vries R. P. Conesa A. de Bekker C. Talon M. Menke H. H. (2007). Spatial differentiation in the vegetative mycelium of *Aspergillus niger*. *Eukaryot. Cell* 6 2311–2322. 10.1128/EC.00244-07 17951513 PMC2168252

[B35] Li H. X. Lu Z. M. Zhu Q. Gong J. S. Geng Y. Shi J. S. (2017). Comparative transcriptomic and proteomic analyses reveal a FluG-mediated signaling pathway relating to asexual sporulation of *Antrodia camphorata*. *Proteomics* 17 17–18. 10.1002/pmic.201700256 28792668

[B36] Li T. Iwaki H. Fu R. Hasegawa Y. Zhang H. Liu A. (2006). α-amino-β-carboxymuconic-𝜀-semialdehyde decarboxylase (ACMSD) is a new member of the amidohydrolase superfamily. *Biochemistry* 45 6628–6634. 10.1021/bi060108c 16716073

[B37] Li W. Cowley A. Uludag M. Gur T. McWilliam H. Squizzato S. (2015). The EMBL-EBI bioinformatics web and programmatic tools framework. *Nucleic Acids Res.* 43 W580–W584. 10.1093/nar/gkv279 25845596 PMC4489272

[B38] Lohkamp B. Andersen B. Piškur J. Dobritzsch D. (2006). The crystal structures of dihydropyrimidinases reaffirm the close relationship between cyclic amidohydrolases and explain their substrate specificity. *J. Biol. Chem.* 281 13762–13776. 10.1074/jbc.M513266200 16517602

[B39] Mah J. H. Yu J. H. (2006). Upstream and downstream regulation of asexual development in *Aspergillus fumigatus*. *Eukaryot. Cell* 5 1585–1595. 10.1128/EC.00192-06 17030990 PMC1595350

[B40] Margelis S. D’Souza C. Small A. J. Hynes M. J. Adams T. H. Davis M. A. (2001). Role of glutamine synthetase in nitrogen metabolite repression in *Aspergillus nidulans*. *J. Bacteriol.* 183 5826–5833. 10.1128/JB.183.20.5826-5833.2001 11566979 PMC99658

[B41] Markina-Iñarrairaegui A. Etxebeste O. Herrero-García E. Araújo-Bazán L. Fernández-Martínez J. Flores J. A. (2011). Nuclear transporters in a multinucleated organism: functional and localization analyses in *Aspergillus nidulans*. *Mol. Biol. Cell* 22 3874–3886. 10.1091/mbc.E11-03-0262 21880896 PMC3192866

[B42] Márquez-Fernández O. Trigos Á Ramos-Balderas J. L. Viniegra-González G. Deising H. B. Aguirre J. (2007). Phosphopantetheinyl transferase CfwA/NpgA is tequired for *Aspergillus nidulans* secondary metabolism and asexual development. *Eukaryot. Cell* 6 710–720. 10.1128/EC.00362-06 17277172 PMC1865657

[B43] Mathis R. Gamas P. Meyer Y. Cullimore J. V. (2000). The presence of GSI-like genes in higher plants: support for the paralogous evolution of GSI and GSII genes. *J. Mol. Evol.* 50 116–122. 10.1007/s002399910013 10684345

[B44] Mathis R. Grosjean C. de Billy F. Huguet T. Gamas P. (1999). The early nodulin gene MtN6 is a novel marker for events preceding infection of *Medicago truncatula* roots by *Sinorhizobium meliloti*. *Mol. Plant Microbe Interact.* 12 544–555. 10.1094/MPMI.1999.12.6.544 10356802

[B45] Murray D. S. Chinnam N. Tonthat N. K. Whitfill T. Wray LV Jr. Fisher S. H. (2013). Structures of the *Bacillus subtilis* glutamine synthetase dodecamer reveal large intersubunit catalytic conformational changes linked to a unique feedback inhibition mechanism. *J. Biol. Chem.* 288 35801–35811. 10.1074/jbc.M113.519496 24158439 PMC3861631

[B46] Nam S. H. Park H. S. Kim H. S. (2005). Evolutionary relationship and application of a superfamily of cyclic amidohydrolase enzymes. *Chem. Rec.* 5 298–307. 10.1002/tcr.20057 16211624

[B47] Nguyen T. T. Fedorov A. A. Williams L. Fedorov E. V. Li Y. Xu C. (2009). The mechanism of the reaction catalyzed by uronate isomerase illustrates how an isomerase may have evolved from a hydrolase within the amidohydrolase superfamily. *Biochemistry* 48 8879–8890. 10.1021/bi901046x 19678710 PMC2773443

[B48] Nguyen Ba A. N. Pogoutse A. Provart N. Moses A. M. (2009). NLStradamus: a simple hidden Markov model for nuclear localization signal prediction. *BMC Bioinform.* 10:202. 10.1186/1471-2105-10-202 19563654 PMC2711084

[B49] Nicholas K. B. Nicholas H. B. Jr. Deerfield D. W. (1997). GeneDoc: analysis and visualization of genetic variation. *EMBnet J.* 4 1–4.

[B50] Nielsen H. (2017). “Predicting secretory proteins with signalP,” in *Protein Function Prediction* 1st Edn ed. Kihara D. (New York, NY: Humana Press) 59–73. 10.1007/978-1-4939-7015-5_6 28451972

[B51] Nielsen M. L. Nielsen J. B. Rank C. Klejnstrup M. L. Holm D. K. Brogaard K. H. (2011). A genome-wide polyketide synthase deletion library uncovers novel genetic links to polyketides and meroterpenoids in *Aspergillus nidulans*. *FEMS Microbiol. Lett.* 321 157–166. 10.1111/j.1574-6968.2011.02327.x 21658102

[B52] Ogawa M. Tokuoka M. Jin F. J. Takahashi T. Koyama Y. (2010). Genetic analysis of conidiation regulatory pathways in koji-mold *Aspergillus oryzae*. *Fungal Genet. Biol.* 47 10–18. 10.1016/j.fgb.2009.10.004 19850144

[B53] Peñalva M. A. (2005). Tracing the endocytic pathway of *Aspergillus nidulans* with FM4-64. *Fungal Genet. Biol.* 42 963–975. 10.1016/j.fgb.2005.09.004 16291501

[B54] Pontecorvo G. Roper J. A. Hemmons L. M. Macdonald K. D. Bufton A. W. J. (1953). The genetics of *Aspergillus nidulans*. *Advan. Genet.* 5 141–238. 10.1016/S0065-2660(08)60408-3 13040135

[B55] R Core Team. (2008). *R: A Language and Environment for Statistical Computing*. Vienna: R Foundation for Statistical Computing.

[B56] Rechsteiner M. Rogers S. W. (1996). PEST sequences and regulation by proteolysis. *Trends Biochem. Sci.* 21 267–271. 10.1016/S0968-0004(96)10031-1 8755249

[B57] Rexer H. U. Schäberle T. Wohlleben W. Engels A. (2006). Investigation of the functional properties and regulation of three glutamine synthetase-like genes in *Streptomyces coelicolor* A3(2). *Arch. Microbiol.* 186 447–458. 10.1007/s00203-006-0159-8 16932908

[B58] Rodriguez-Urra A. B. Jimenez C. Nieto M. I. Rodriguez J. Hayashi H. Ugalde U. (2012). Signaling the induction of sporulation involves the interaction of two secondary metabolites in *Aspergillus nidulans*. *ACS Chem. Biol.* 7 599–606. 10.1021/cb200455u 22234162

[B59] Sastry G. M. Adzhigirey M. Day T. Annabhimoju R. Sherman W. (2013). Protein and ligand preparation: parameters, protocols, and influence on virtual screening enrichments. *J. Comput. Aided Mol. Des.* 27 221–234. 10.1007/s10822-013-9644-8 23579614

[B60] Schindelin J. Arganda-Carreras I. Frise E. Kaynig V. Longair M. Pietzsch T. (2012). Fiji: an open-source platform for biological-image analysis. *Nat. Methods* 9 676–682. 10.1038/nmeth.2019 22743772 PMC3855844

[B61] Schumacher J. Simon A. Cohrs K. C. Traeger S. Porquier A. Dalmais B. (2015). The VELVET complex in the gray mold fungus *Botrytis cinerea*: impact of BcLAE1 on differentiation, secondary metabolism, and virulence. *Mol. PlantMicrobe Interact.* 28 659–674. 10.1094/MPMI-12-14-0411-R 25625818

[B62] Seibert C. M. Raushel F. M. (2005). Structural and catalytic diversity within the amidohydrolase superfamily. *Biochemistry* 44 6383–6391. 10.1021/bi047326v 15850372

[B63] Shelley J. C. Cholleti A. Frye L. L. Greenwood J. R. Timlin M. R. Uchimaya M. (2007). Epik: a software program for pKa prediction and protonation state generation for drug-like molecules. *J. Comput. Aided Mol. Des.* 21 681–691. 10.1007/s10822-007-9133-z 17899391

[B64] Shivakumar D. Williams J. Wu Y. Damm W. Shelley J. Sherman W. (2010). Prediction of absolute solvation free energies using molecular dynamics free energy perturbation and the OPLS force field. *J. Chem. Theory Comput.* 6 1509–1519. 10.1021/ct900587b 26615687

[B65] Silva L. S. Seabra A. R. Leitão J. N. Carvalho H. G. (2015). Possible role of glutamine synthetase of the prokaryotic type (GSI-like) in nitrogen signaling in *Medicago truncatula*. *Plant Sci.* 240 98–108. 10.1016/j.plantsci.2015.09.001 26475191

[B66] Sonnhammer E. L. von Heijne G. Krogh A. (1998). “A hidden Markov model for predicting transmembrane helices in protein sequences,” in *Proceedings of the Sixth International Conference on Intelligent Systems for Molecular Biology* eds Glasgow J. Littlejohn T. Major F. Lathrop R. Sankoff D. Sensen C. (Menlo Park, CA: AAAI Press) 175–182. 9783223

[B67] Strong M. Mallick P. Pellegrini M. Thompson M. J. Eisenberg D. (2003). Inference of protein function and protein linkages in *Mycobacterium tuberculosis* based on prokaryotic genome organization: a combined computational approach. *Genome Biol.* 4:R59. 10.1186/gb-2003-4-9-r59 12952538 PMC193659

[B68] Szewczyk E. Nayak T. Oakley C. E. Edgerton H. Xiong Y. Taheri-Talesh N. (2006). Fusion PCR and gene targeting in *Aspergillus nidulans*. *Nat. Protoc.* 1 3111–3120. 10.1038/nprot.2006.405 17406574

[B69] Takeo M. Ohara A. Sakae S. Okamoto Y. Kitamura C. Kato D. (2013). Function of a glutamine synthetase-like protein in bacterial aniline oxidation via γ-Glutamylanilide. *J. Bacteriol.* 195 4406–4414. 10.1128/JB.00397-13 23893114 PMC3807463

[B70] Takeshita N. Higashitsuji Y. Konzack S. Fischer R. (2008). Apical sterol-rich membranes are essential for localizing cell end markers that determine growth directionality in the filamentous fungus *Aspergillus nidulans*. *Mol. Biol. Cell* 19 339–351. 10.1091/mbc.E07-06-0523 18003978 PMC2174190

[B71] Tilburn J. Scazzocchio C. Taylor G. G. Zabicky-Zissman J. H. Lockington R. A. Davies R. W. (1983). Transformation by integration in *Aspergillus nidulans*. *Gene* 26 205–221. 10.1016/0378-1119(83)90191-9 6368319

[B72] Trevaskis B. Wandrey M. Colebatch G. Udvardi M. K. (2002). The soybean GmN6L gene encodes a late nodulin expressed in the infected zone of nitrogen-fixing nodules. *Mol. Plant Microbe Interact.* 15 630–636. 10.1094/MPMI.2002.15.7.630 12118878

[B73] Vinck A. de Bekker C. Ossin A. Ohm R. A. de Vries R. P. Wösten H. A. B. (2011). Heterogenic expression of genes encoding secreted proteins at the periphery of *Aspergillus niger* colonies. *Environ. Microbiol.* 13 216–225. 10.1111/j.1462-2920.2010.02322.x 20722697

[B74] Walker M. C. van der Donk W. A. (2016). The many roles of glutamate in metabolism. *J. Ind. Microbiol. Biotechnol.* 43 419–430. 10.1007/s10295-015-1665-y 26323613 PMC4753154

[B75] Wang F. Krijgsheld P. Hulsman M. de Bekker C. Müller W. H. Reinders M. (2015). FluG affects secretion in colonies of *Aspergillus niger*. *Antonie Van Leeuwenhoek* 107 225–240. 10.1007/s10482-014-0321-2 25370014

[B76] Waterhouse A. M. Procter J. B. Martin D. M. A. Clamp M. Barton G. J. (2009). Jalview version 2–a multiple sequence alignment editor and analysis workbench. *Bioinformatics* 25 1189–1191. 10.1093/bioinformatics/btp033 19151095 PMC2672624

[B77] Yager L. N. Lee H. O. Nagle D. L. Zimmerman J. E. (1998). Analysis of *fluG* mutations that affect light-dependent conidiation in *Aspergillus nidulans*. *Genetics* 149 1777–1786. 9691036 10.1093/genetics/149.4.1777 PMC1460255

[B78] Yang L. Ukil L. Osmani A. Nahm F. Davies J. De Souza C. P. C. (2004). Rapid production of gene replacement constructs and generation of a green fluorescent protein-tagged centromeric marker in *Aspergillus nidulans*. *Eukaryot. Cell* 3 1359–1362. 10.1128/EC.3.5.1359-1362.2004 15470263 PMC522605

[B79] Yao X. He W. Lu C. D. (2011). Functional characterization of seven γ-Glutamylpolyamine synthetase genes and the bauRABCD locus for polyamine and β-Alanine utilization in *Pseudomonas aeruginosa* PAO1. *J. Bacteriol.* 193 3923–3930. 10.1128/JB.05105-11 21622750 PMC3147493

[B80] Yu J. H. Mah J. H. Seo J. A. (2006). Growth and developmental control in the model and pathogenic *Aspergilli*. *Eukaryot. Cell* 5 1577–1584. 10.1128/EC.00193-06 17030989 PMC1595332

