# Supplementary material for: The Early Asexual Development Regulator fluG Codes for a Putative Bifunctional Enzyme
Source: Front Microbiol. 2019 Apr 17;10:778. doi: 10.3389/fmicb.2019.00778 (PMC6478659; doi:10.3389/fmicb.2019.00778)
Supplement: Supplementary file 1 [file Data_Sheet_1.docx]

Supplementary Material

# The Early Asexual Development Regulator *fluG* Codes for a Putative Bifunctional Enzyme

Mikel Iradi-Serrano, Leire Tola-García, Marc S. Cortese and Unai Ugalde^*^

*** Correspondence:**Unai Ugalde
unaiona.ugalde@ehu.eus

# Supplementary Figures and Tables

## Supplementary Figures

##
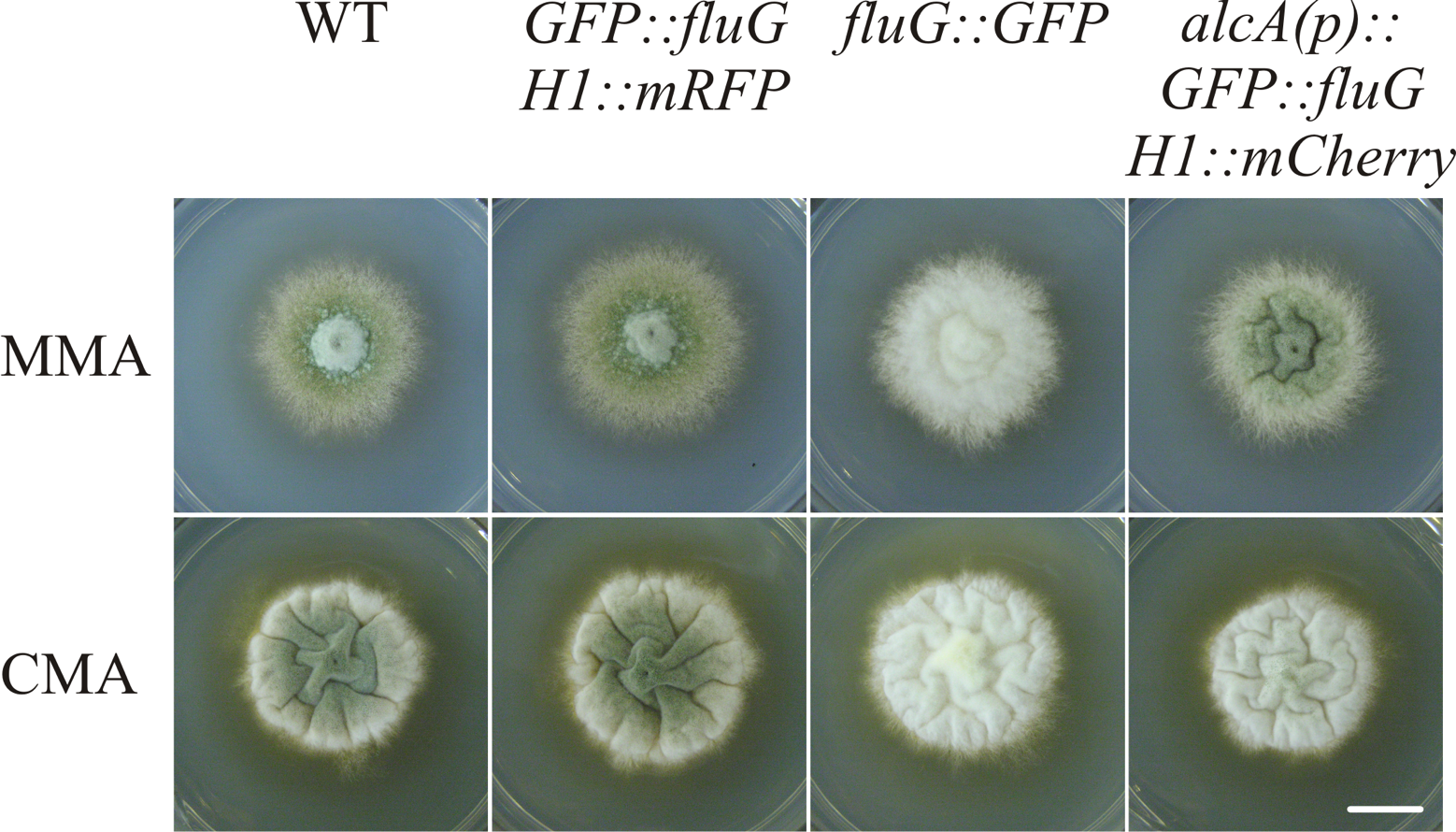


## Supplementary Figure 1. Phenotypes of GFP-tagged strains grown in minimal and complete media (MMA and CMA) supplemented with glucose. N-terminally tagged *fluG* shows a wild type-like (WT) phenotype in both MMA and CMA; conversely, the C-terminally tagged *fluG* exhibits a fluffy phenotype. The *alcA(p)::GFP::fluG* strain presents a partially conidial phenotype in MMA and very low conidial phenotype in CMA. Scale bar = 1 cm

##
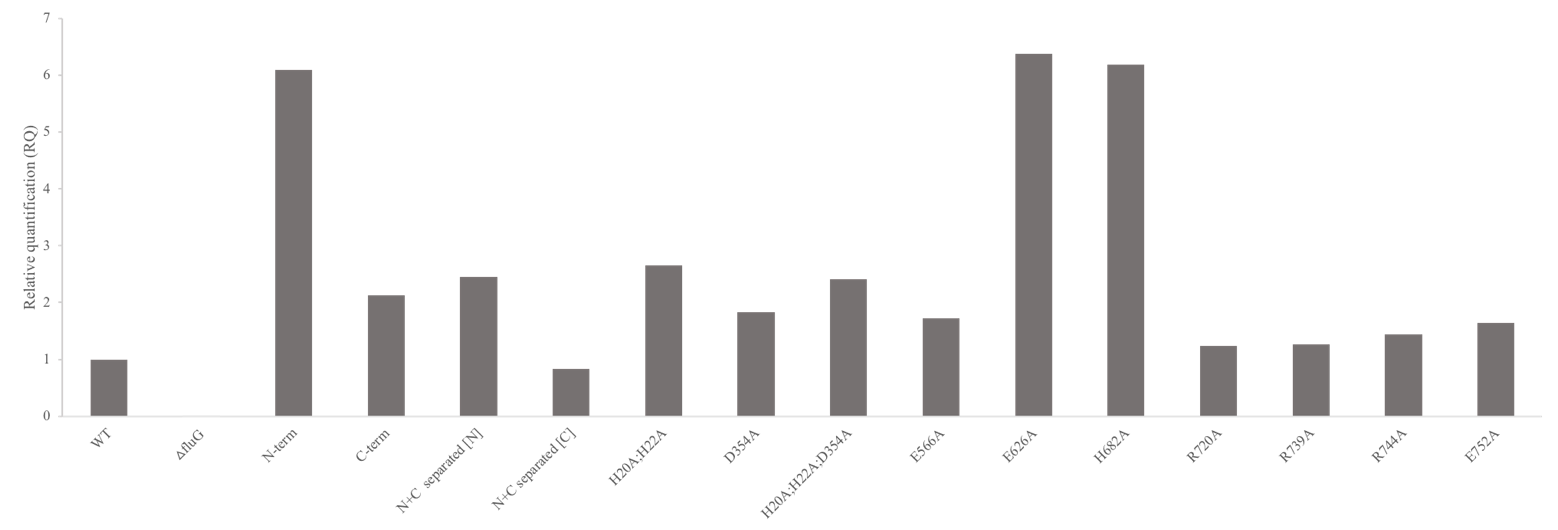
Supplementary Figure 2. *fluG* expression values for each strain in MMA are shown in the plot. All samples were taken after 18 h of vegetative growth. Acronyms are referred as follows: WT, wild type; *ΔfluG*, *fluG* null mutant; N-term, (1-406) region; C-term, (427-865) region; N+C separated [N]/N+C separated [C], N- and C-terminal separated region mutant; H20A-H22A/D354A/H20A-H22A-D354A, N-terminal point mutants; E566A/E626A/H682A/R720A/ R739A/R744A/E752A, C-terminal point mutants.

## Supplementary Tables

**Supplementary Table 1.** Strains and plasmids used in this study.

| Strain | Genotype | Source |
| --- | --- | --- |
| TTA127.4 | *yA2; ΔfluG::trpC^+^; pabaA1;trpC801; veA1* | (Lee and Adams, 1994) |
| TN02A3 | *pyrG89; argB2; pyroA4; ΔnkuA::argB^+^; veA1* | (Nayak et al., 2006) |
| CRO1 | *cfwA2; yA2; pabaA1; veA1* | (Márquez-Fernández et al., 2007) |
| BD688 | *pyrG89; argB2; pyroA4; ΔausA::pyrG^+^; ΔnkuA::argB^+^; veA1* | This study |
| WIM 126 | *yA2*; *pabaA1*; *veA+* | (Butnick et al., 1984) |
| FGSC A#1155 | *pyrG89*; *ΔnkuA*::*bar*; *pyroA4*; *veA1* | Fungal Genetics Stock Center  (McCluskey et al., 2010) |
| BD824 | *pyrG89*; *ΔnkuA*::*bar*; *pyroA4*; *veA+* | This study (Cross between WIM126 x FGSC A#1155) |
| BD969 | *pyrG89; ΔfluG::pyrG; ΔnkuA::bar; pyroA4; veA+* | This study |
| BD970 | *pyrG89; fluG(1-406); ΔnkuA::bar; pyroA4; veA+* | This study |
| BD971 | *pyrG89; fluG(427-865); ΔnkuA::bar; pyroA4; veA+* | This study |
| BD972 | *pyrG89; fluG(H20A;H22A); ΔnkuA::bar; pyroA4; veA+* | This study |
| BD975 | *pyrG89; fluG::GFP::pyrG; ΔnkuA::bar; pyroA4; veA+* | This study |
| BD1145 | *pyrG89; ΔfluG::LSEI_0440::fluG(407-865); ΔnkuA::bar; pyroA4; veA+* | This study |
| BD1194 | *pyrG89; fluG(1-406); ΔnkuA::bar; pyroA4; fluG(427-865)::pyroA; veA+* | This study |
| BD1195 | *pyrG89; fluG(D354A);ΔnkuA::bar; pyroA4; veA+* | This study |
| BD1196 | *pyrG89; fluG(H20A;H22A;D354A);ΔnkuA::bar; pyroA4; veA+* | This study |
| BD1197 | *pyrG89*; *fluG(E566A)*;*ΔnkuA*::*bar*; *pyroA4*; *veA+* | This study |
| BD1198 | *pyrG89*; *fluG(E626A)*;*ΔnkuA*::*bar*; *pyroA4*; *veA+* | This study |
| BD1199 | *pyrG89*; *fluG(H682A)*; *ΔnkuA*::*bar*; *pyroA4*; *veA+* | This study |
| BD1200 | *pyrG89*; *fluG(R720A)*; *ΔnkuA*::*bar*; *pyroA4*; *veA+* | This study |
| BD1201 | *pyrG89*; *fluG(R739A)*; *ΔnkuA*::*bar*; *pyroA4*; *veA+* | This study |
| BD1202 | *pyrG89*; *fluG(R744A)*; *ΔnkuA*::*bar*; *pyroA4*; *veA+* | This study |
| BD1203 | *pyrG89*; *fluG(E752A)*; *ΔnkuA*::*bar*; *pyroA4*; *veA+* | This study |
| BD1206 | *pyrG89; GFP::fluG; ΔnkuA::bar; pyroA4; hhoA::mRFP::pyrG; veA+* | This study |
| BD1207 | *pyrG89; alcA(p)::GFP::fluG::pyrG; ΔnkuA::bar; pyroA4; hhoA::mCherry::pyroA; veA+* | This study |
| BD1229 | *pyrG89; ΔfluG::PA5508; ΔnkuA::bar; pyroA4; veA+* | This study |
| BD1231 | *pyrG89; ΔfluG::fluG(1-426)::PA5508; ΔnkuA::bar; pyroA4; veA+* | This study |
| BD1233 | *pyrG89; ΔfluG::LSEI_0440::fluG(407-426)::PA5508; ΔnkuA::bar; pyroA4; veA+* | This study |
| Plasmid | Construction | Source |
| pNT5 | *alcA(p)::GFP*, for N-terminal fusion of GFP to proteins; contains *N. crassa pyr4*; 0.7 kb *tea* fragment with AscI and PacI sites. | Takeshita et al., 2008 |
| pMI2 | 1 kb *fluG* N-terminal fragment with AscI and BamHI sites in pNT5 | This study |

**Supplementary Table 2.** Oligonucleotides used in this study. The restriction sites are underlined. Oligonucleotides marked with asterisk (*) have been previously published by Ruger-Herreros et al. (2011).

| Oligonucleotide | Sequence (5´→3´) | Designed for |
| --- | --- | --- |
| fluG-PP1-F | CGACCAAAGACAACGCTATTGACCGC | 5´-UTR of *fluG* |
| fluG-PP2-R | CATGGCGATGAACCAGCAAACTAAAGG | 5´-UTR of *fluG* |
| fluG-SMP1-F | CCTTTAGTTTGCTGGTTCATCGCCATGACCGGTCGCCTCAAACAATGCTCT | *ΔfluG* |
| fluG-GFP2-R | GGAAATATTGTGAATACGCTCAGGAGAAAGATTAGACTCAGTCTGAGAGGAGGCACTGATGCG | *ΔfluG* |
| fluG-GSP3-F | TGAGTCTAATCTTTCTCCTGAGCGTATTCACAATATTTCC | 3´-UTR of *fluG* |
| fluG-GSP4-R | CCTACAAACTCTGCTGAAGACGTCGATTCC | 3´-UTR of *fluG* |
| fluG-geneSP-F | ATGGCCACTCTCTCTTCACTCCGTCATC | *fluG* sequencing |
| fluG(1-406)-R | CGCTCAGGAGAAAGATTAGACTCAAAGCCGGTTTGAATTGTGG | N-terminal *fluG* |
| prom-fluG(427-865)-F | CCTTTAGTTTGCTGGTTCATCGCCATGCGTATCTCGTCAACTGATCTGC | C-terminal *fluG* |
| FarPP1_pyroA-F | CGAGTGGGATGGAAATACTGAGCGTCC | N+C-terminal separated |
| GSP4-pyroA-R | CCCTACGAACCATGGCATTCCTCATTCAGC | N+C-terminal separated |
| FarPP2-pyroA-fluGPP1-R | CGCGGTCAATAGCGTTGTCTTTGGTCGTCCGATGGCAATTTACCTGCCG | N+C-terminal separated |
| fluGGSP4-NrPP1-pyroA-R | GGAATCGACGTCTTCAGCAGAGTTTGTAGGATGGAGGTTTAACTCCGGTCAGG | N+C-terminal separated |
| AscI_fluG(-ATG)-F | GGGGCGCGCCCGCCACTCTCTCTTCACTCC | pMI2 |
| BamHI_fluG-R | CGGGATCCCCATAACAACCGAGTGCTGGG | pMI2 |
| H20AH22A-F | CATCGACAACGCTGCTGCCAACCTCCTCTC | H20A-H22A mutant |
| H20AH22A-R | GAGAGGAGGTTGGCAGCAGCGTTGTCGATG | H20A-H22A mutant |
| D354A-F | GTTATGGAGTACCGCTGGGCATTTTTTCC | D354A mutant |
| D354A-R | GGAAAAAATGCCCAGCGGTACTCCATAAC | D354A mutant |
| E566A-F | GCGGCTTTGAGATTGCAGTCGTTTTCTTGAAGC | E566A mutant |
| E566A-R | GCTTCAAGAAAACGACTGCAATCTCAAAGCCGC | E566A mutant |
| E626A-F | CAACAATTCCATGCCGCGTCCGCCCCTGGC | E626A mutant |
| E626A-R | GCCAGGGGCGGACGCGGCATGGAATTGTTG | E626A mutant |
| H682A-F | GCACGGCGTCCGCCGCGCACGTTTCC | H682A mutant |
| H682A-R | GGAAACGTGCGCGGCGGACGCCGTGC | H682A mutant |
| R720A-F | GCAAGCTACGACGCCGTTAAGTCGGGTATTTGG | R720A mutant |
| R720A-R | CCAAATACCCGACTTAACGGCGTCGTAGCTTGC | R720A mutant |
| R739A-F | GGGCACCCAGAACGCTGAGGCGCCTATTCG | R739A mutant |
| R739A-R | CGAATAGGCGCCTCAGCGTTCTGGGTGCCC | R739A mutant |
| R744A-F | CGTGAGGCGCCTATTGCCAAGATCTCACCAGG | R744A mutant |
| R744A-R | CCTGGTGAGATCTTGGCAATAGGCGCCTCACG | R744A mutant |
| E752A-F | CTCACCAGGCCATTGGGCAATCAAGTCTCTTGACG | E752A mutant |
| E752A-R | CGTCAAGAGACTTGATTGCCCAATGGCCTGGTGAG | E752A mutant |
| 440-F | GTTTGCTGGTTCATCGCCATGGACGACTTATCTGAATTTG | LSEI_0440+C-term |
| 440-R | CATTTAACTCATAGACGCGCAGTTCACGCTCTTGATGATAAAGC | LSEI_0440+C-term |
| CTP-F | GAACTGCGCGTCTATGAGTTAAATGAACAACC | LSEI_0440+C-term |
| NTP_PA5508-F | CCA GAC GGT CTC CGT GAA CCG CCT GCA GCC GGT ACG | N-terminal+PA5508 and LSEI_0440+PA5508 |
| NTP_PA5508-R | GGC GGT TCA CGG AGA CCG TCT GGT GCC CAG ACG | N-terminal+PA5508 and LSEI_0440+PA5508 |
| Prom-fluG_PA5508-F | GTT TGC TGG TTC ATC GCC ATG GTG AAC CGC CTG CAG CCG G | PA5508 mutant |
| PA5508_Term-fluG-R | CGC TCA GGA GAA AGA TTA GAC TCA GTA GAG CTC GGC ATA GTG GC | PA5508 mutant |
| benA_qPCR-F | AGATGCGCAACATCCAGAGC | *benA* expression in qPCR |
| benA_qPCR-R | CTGGTACTCGGAGACGAGATCG | *benA* expression in qPCR |
| fluG(N-term)_qPCR-F | GCACTCGGTTGTTATGGAG | *fluG* N-terminal expression in qPCR |
| fluG(N-term)_qPCR-R | TCCACGAAAACCTTTTCCAG | *fluG* N-terminal expression in qPCR |
| fluG(C-term)_qPCR-F | CTCGAAGAAATCGCCGAAAC | *fluG* C-terminal expression in qPCR (*) |
| fluG(C-term)_qPCR-R | CTCGGCATGGAATTGTTGAA | *fluG* C-terminal expression in qPCR (*) |

**Supplementary Table 3.** FluG orthologs present in all the characterized Aspergilli in Ensembl Fungi database.

| **Aspergillus species** | **Ensembl gene entry** | **Identity (%)** |
| --- | --- | --- |
| Aspergillus calidoustus | ASPCAL10313 | 78.50 |
| Aspergillus cristatus | SI65_06388 | 68.55 |
| Aspergillus flavus | AFLA_101920 | 69.94 |
| Aspergillus fumigatus | AFUA_3G07140 | 69.36 |
| Aspergillus lentulus | ALT_2048 | 69.60 |
| Aspergillus luchuensis | RIB2604_01900210 | 70.17 |
| Aspergillus niger | An14g03390 | 70.29 |
| Aspergillus nomius NRRL 13137 | ANOM_010302 | 69.48 |
| Aspergillus ochraceoroseus | AOCH_000790 | 76.30 |
| Aspergillus oryzae | AO090020000217 | 69.94 |
| Aspergillus parasiticus SU-1 | P875_00108952 | 70.29 |
| Aspergillus ruber CBS 135680 | EURHEDRAFT_530090 | 67.98 |
| Aspergillus terreus | ATEG_01762 | 60.12 |
| Aspergillus udagawae | AUD_4531 | 69.94 |

**Supplementary Table 4.** Mean values and standard deviations (in brackets) of conidia (C) and dry cell mass (DCM) measurements in MMA and CMA.

|  | MMA | | CMA | |
| --- | --- | --- | --- | --- |
|  | C/Area (^millions^/_cm2_) | DCM/Area (^mg^/_cm2_) | C/Area (^millions^/_cm2_) | DCM/Area (^mg^/_cm2_) |
| WT; *veA1* | 63 (14) | 2.2 (0.2) | 101 (4) | 4.8 (0.1) |
| *ΔausA*; *veA1* | 66 (7) | 2.4 (0.4) | 104.9 (9.4) | 5.5 (0.3) |

**Supplementary Table 5.** Mean values and standard deviations (in brackets) of conidia (C) and dry cell mass (DCM) measurements in MMA and CMA.

|  | MMA | | CMA | |
| --- | --- | --- | --- | --- |
|  | C/Area (^millions^/_cm2_) | DCM/Area (^mg^/_cm2_) | C/Area (^millions^/_cm2_) | DCM/Area (^mg^/_cm2_) |
| WT | 4.1 (0.9) | 2.2 (0.4) | 21 (6) | 4.8 (0.2) |
| *ΔfluG* | 0.02 (0.01) | 3.3 (0.5) | 0.003 (0.003) | 4.4 (0.2) |
| N-terminal | 0.03 (0.02) | 2.8 (0.4) | 0.009 (0.008) | 4.1 (0.4) |
| C-terminal | 5 (1) | 3.2 (0.3) | 4 (1) | 4.7 (0.6) |
| N+C separated | 5 (1) | 2.4 (0.4) | 9 (3) | 4.4 (0.6) |
| WT | 5.0 (0.3) | 2.4 (0.3) | 19 (3) | 4.9 (0.4) |
| *ΔfluG* | 0.03 (0.02) | 3.0 (0.4) | 0.005 (0.004) | 4.6 (0.4) |
| H20A-H22A | 2.8 (0.6) | 2.7 (0.2) | 11 (1) | 4.4 (0.7) |
| D354A | 2.9 (0.3) | 3.5 (0.5) | 10.2 (0.7) | 4.4 (0.4) |
| H20A-H22A-D354A | 2.7 (0.1) | 3.2 (0.3) | 5.1 (0.7) | 5.3 (0.7) |
| E566A | 0.5 (0.3) | 3.7 (0.4) | 0.006 (0.007) | 4.9 (0.7) |
| E626A | 0.4 (0.3) | 3.7 (0.5) | 0.003 (0.003) | 4.6 (0.6) |
| H682A | 0.8 (0.3) | 3.6 (0.9) | 0.012 (0.008) | 4.7 (0.2) |
| R720A | 1.0 (0.6) | 3.4 (0.4) | 0.004 (0.004) | 4.5 (0.3) |
| R739A | 1.0 (0.3) | 3.4 (0.5) | 1.2 (0.8) | 6 (1) |
| R744A | 0.10 (0.07) | 3.1 (0.3) | 0 (0) | 4.0 (0.3) |
| E752A | 0.1 (0.1) | 2.7 (0.4) | 0.006 (0.008) | 4.1 (0.5) |
| WT | 4 (1) | 2.7 (0.3) | 22 (5) | 4.7 (0.6) |
| *ΔfluG* | 0.03 (0.02) | 3.3 (0.1) | 0.0061 (0.0003) | 4.2 (0.4) |
| LSEI_0440+ C-term | 13 (2) | 2.1 (0.2) | 23 (5) | 3.5 (0.8) |
| N-term+ PA5508 | 6 (2) | 2.5 (0.3) | 24 (9) | 4.6 (0.2) |
| LSEI_0440+  PA5508 | 5 (2) | 2.6 (0.4) | 19 (5) | 4.8 (0.2) |
| PA5508 | 5 (1) | 2.3 (0.2) | 11 (4) | 4.7 (0.3) |

**References**

Butnick, N.Z., Yager, L.N., Kurtz, M.B., and Champe, S.P. (1984). Genetic Analysis of Mutants of *Aspergillus nidulans* Blocked at an Early Stage of Sporulation. *J Bacteriol* 160(2)**,** 541-545.

Lee, B.N., and Adams, T.H. (1994). The *Aspergillus nidulans* *fluG* gene is required for production of an extracellular developmental signal and is related to prokaryotic glutamine synthetase I. *Genes Dev.* 8(6)**,** 641-651. doi: 10.1101/gad.8.6.641.

Márquez-Fernández, O., Trigos, Á., Ramos-Balderas, J.L., Viniegra-González, G., Deising, H.B., and Aguirre, J. (2007). Phosphopantetheinyl Transferase CfwA/NpgA Is Tequired for *Aspergillus nidulans* Secondary Metabolism and Asexual Development. *Eukaryot Cell* 6(4)**,** 710-720. doi: 10.1128/EC.00362-06.

McCluskey, K., Wiest, A., and Plamann, M. (2010). The Fungal Genetics Stock Center: a repository for 50 years of fungal genetics research. *J Biosci* 35(1)**,** 119-126. doi: 10.1007/s12038-010-0014-6.

Nayak, T., Szewczyk, E., Oakley, C.E., Osmani, A., Ukil, L., Murray, S.L., et al. (2006). A versatile and efficient gene-targeting system for *Aspergillus nidulans*. *Genetics* 172(3)**,** 1557-1566. doi: genetics.105.052563 [pii];10.1534/genetics.105.052563 [doi].

Ruger-Herreros, C., Rodríguez-Romero, J., Fernández-Barranco, R., Olmedo, M., Fischer, R., Corrochano, L.M., et al. (2011). Regulation of Conidiation by Light in *Aspergillus nidulans*. *Genetics* 188(4)**,** 809-822. doi: 10.1534/genetics.111.130096.
